# Comparative muscle anatomy of the anuran pelvis and hindlimb in relation to locomotor mode

**DOI:** 10.1111/joa.14122

**Published:** 2024-08-09

**Authors:** Alice Leavey, Christopher T. Richards, Laura B. Porro

**Affiliations:** ^1^ Centre for Integrative Anatomy, Cell and Developmental Biology University College London London UK; ^2^ Structure and Motion Laboratory Royal Veterinary College—Camden Campus, Comparative Biomedical Sciences London UK

**Keywords:** diceCT, dissection, frog, imaging, locomotion, musculoskeletal

## Abstract

Frogs have a highly conserved body plan, yet they employ a diverse array of locomotor modes, making them ideal organisms for investigating the relationships between morphology and locomotor function, in particular whether anatomical complexity is a prerequisite for functional complexity. We use diffusible iodine contrast‐enhanced microCT (diceCT) imaging to digitally dissect the gross muscle anatomy of the pelvis and hindlimbs for 30 species of frogs representing five primary locomotor modes, including the first known detailed dissection for some of the world's smallest frogs, forming the largest digital comparative analysis of musculoskeletal structure in any vertebrate clade to date. By linking musculoskeletal dissections and phylogenetic comparative methods, we then quantify and compare relationships between anatomy and function across over 160 million years of anuran evolution. In summary, we have found that bone lengths and pelvic crest sizes are generally not reliable predictors of muscle sizes, which highlights important implications for future palaeontological studies. Our investigation also presents previously unreported differences in muscle anatomy between frogs specialising in different locomotor modes, including several of the smallest frog hindlimb muscles, which are extremely difficult to extract and measure using traditional approaches. Furthermore, we find evidence of many‐to‐one and one‐to‐many mapping of form to function across the phylogeny. Additionally, we perform the first quantitative analysis of how the degree of muscle separation can differ between frogs. We find evidence that phylogenetic history is the key contributing factor to muscle separation in the pelvis and thigh, while the separation of shank muscles is influenced more strongly by locomotor mode. Finally, our anatomical 3D reconstructions are published alongside this manuscript to contribute towards future research and serve as educational materials.

## Graphical abstract

Scientists at UCL and the Royal Veterinary College have digitally dissected the musculoskeletal anatomy of the pelvis and hindlimbs of 30 frog species using contrast‐enhanced microCT scanning, forming the largest digital anatomical dataset in any vertebrate group to date. The team have discovered previously unreported anatomical differences between frogs specialising in different locomotor modes and have published all of the 3D anatomical reconstructions with detailed annotations, free for anyone to download.

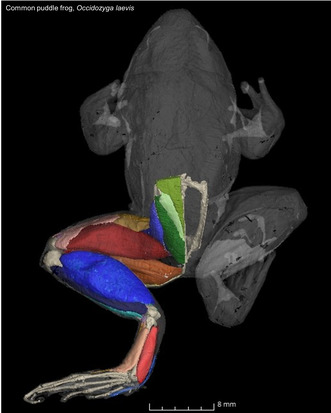

## 1 INTRODUCTION

The complex relationship between musculoskeletal form and function, and how it influences animal behaviour, has posed a major, long‐standing challenge in evolutionary biology. For example, the behaviour and ecology of extinct animals must be inferred from limited fossilised remains; thus, a tight correlation between bone and soft‐tissue anatomy, and how this relates to function, is often assumed (Bates et al., 2021). To better understand the strength of the relationship between form and function in extinct taxa, anatomical characteristics must be measured in living species for which behaviour and ecology are known (Perry & Prufrock, 2018). However, even this approach faces difficulty due to the ability of one trait to influence multiple functions (i.e. ‘one‐to‐many mapping’) and multiple morphological configurations to enable the same function (i.e. ‘many‐to‐one mapping’; Bergmann & McElroy, 2014; Holzman et al., 2011; Moen, 2019; Wainwright et al., 2005). Therefore, both detailed descriptions of how musculoskeletal anatomy varies among species and quantitative tests of how this can impact function must be carried out to fully understand the evolutionary origins of biological niches.

Anura, part of the class Amphibia, are ideal model organisms for tackling this fundamental challenge. Relatively small anatomical differences in their largely conserved body plan enable frogs to respond to various mechanical challenges and inhabit numerous ecological niches (Citadini et al., 2018; Gomes et al., 2009; Leavey et al., 2023; Lires et al., 2016; Moen et al., 2013; Soliz et al., 2017; Tulli et al., 2016; Vidal‐García et al., 2014). For example, frogs use a range of locomotor modes including walking, hopping, jumping, swimming, burrowing and climbing to traverse different terrestrial, aquatic, arboreal and subterranean environments (Wells, 2007). Interspecific variation in pelvic and hindlimb myology has been long assumed to indicate differences in anuran locomotor behaviour (Collings & Richards, 2019; Fabrezi et al., 2014; Nauwelaerts et al., 2007; Ponssa et al., 2018), where a larger muscle indicates higher functional importance, as more energy has been invested into its growth despite the associated physiological and anatomical costs (e.g. daily energy expenditure) (Perry & Prufrock, 2018). Due to similar locomotor and microhabitat requirements, frogs also show repeated independent evolution of similar phenotypes on a global scale, suggesting that there are a limited number of ways in which frogs can respond to selection (Moen, 2019; Moen et al., 2016). This makes them ideal organisms for investigating the relationship between morphology, function, ecology and evolutionary history. However, many gaps in our knowledge of the anuran musculoskeletal system and its relationship to locomotor function remain, as a detailed comparative analysis of muscle anatomy has not been performed across multiple representative species for each primary locomotor mode.

Given that pelvic skeletal structure is one of the key determinants of morphological variation and locomotor performance in frogs (Buttimer et al., 2020; Emerson, 1979, 1982; Jorgensen & Reilly, 2013; Leavey et al., 2023; Pugener & Maglia, 2009; Reilly & Jorgensen, 2011; Soliz et al., 2017), it is likely that pelvic muscles show significant differences in size and shape between frogs specialising in different locomotor modes, yet this has not been quantified across a large taxonomic and functional range. Pelvic muscles have also been observed to vary in the extent to which they insert onto the ilia and urostyle, which could vary with the presence and development of dorsal crests (Emerson, 1979, 1982; Fabrezi et al., 2014; Jorgensen & Reilly, 2013; Ponssa et al., 2018; Přikryl et al., 2009; Reilly & Jorgensen, 2011). The subtle differences in bone shape could alter the origins or insertions of muscles enough to change their moment arms (Collings & Richards, 2019), but this variation has only been quantified thoroughly across one genus (Ponssa et al., 2018). Furthermore, several studies have suggested that differences in muscle size may not be reflected in the size of their associated long bones, presenting challenges for palaeontological studies (Bates et al., 2021; Perry & Prufrock, 2018; Rabey et al., 2015).

Several studies have investigated how total hindlimb mass is associated with locomotor performance in frogs (Choi et al., 2003; Marsh & John‐Alder, 1994; Moen, 2019), but there are no studies comparing the relative proportions of total muscle mass within each hindlimb segment. Jumpers and swimmers may invest more strongly in shank muscles, as the sizes of ankle extensors are linked to jump force and propulsive foot rotations during swimming (Astley, 2016; Gillis & Biewener, 2000; James et al., 2005). Since backward‐burrowing frogs need to scoop dense substrate with their feet (Emerson, 1976), burrowers may invest more in proximal foot muscles than non‐burrowers. It is not known whether this still applies to frogs which dig using their forelimbs and heads, known as ‘forward burrowers’ (Emerson, 1976), which have evolved more recently. Furthermore, relatively few studies compare the gross architectural properties of more than just the largest muscles (Nauwelaerts et al., 2007; Přikryl et al., 2009; Vera et al., 2022), despite the knowledge that muscles are not mechanically independent ‐ the function of a single muscle often depends on the configuration of joints and therefore the actions of other muscles (Collings & Richards, 2019). Where the complete musculature of multiple species has been described comprehensively, comparisons are largely qualitative (Přikryl et al., 2009). One of the biggest knowledge gaps remaining is how investment into each group of post‐sacral muscles with similar functions (herein referred to as ‘functional muscle groups’) differs between locomotor modes. Additionally, anuran pelvic and hindlimb muscles have been observed to vary in the degree of separation (Dunlap, 1960; Přikryl et al., 2009), but this is yet to be quantified. For example, relative to other species, the highly specialised aquatic frog *Xenopus laevis* has a low degree of muscle separation in the thigh, but considerable separation of the pelvic muscle iliacus externus (Porro & Richards, 2017). Muscle belly separation can be in the form of entirely distinct heads, which can also remain attached at one end of the muscle, as well in the form of a tendinous insertion within the muscle body, known as intramuscular separation (Collings & Richards, 2019; Přikryl et al., 2009). Both of these types of muscle separation are thought to increase the functional range of a limb and are therefore likely to have consequences for locomotion (Collings & Richards, 2019; Gans & Bock, 1965; Roberts, 2002), but the drivers of muscle separation have not yet been analysed. In *X. laevis*, for instance, it is unknown whether the amount of muscle separation in each part of its body reflects its locomotor specialisation, or its basal position in the anuran phylogeny.

Traditionally, physical dissections have been used to record and compare musculoskeletal anatomy (Duellman & Trueb, 1986; Dunlap, 1960; Emerson, 1979; Nauwelaerts et al., 2007; Přikryl et al., 2009). There are several limitations associated with this invasive technique. Primarily, its destructive nature means that data collection is not repeatable and is therefore largely unsuitable for collecting data from museum specimens, closing off a vast source of potential knowledge. Damage makes the 3D musculoskeletal topology almost impossible to preserve and analyse. This makes modelling the complexity of 3D muscle pathways challenging, especially for muscles which pass through or wrap around other structures. Identifying muscle origins, insertions and lines of action is crucial for functional analyses as these variables determine to how a muscle contributes to the production of joint torque (Collings et al., 2022). Additionally, data can be easily lost, particularly for fragile or small structures, or those with large attachments, such as the hip muscle gemellus, which is difficult to separate from the bone's surface intact (Figure S1).

Diffusible iodine‐based contrast‐enhanced computed microtomography (diceCT) has recently made possible the non‐destructive, high‐resolution digital dissection of soft tissues, with preservation of the 3D topology in vertebrates (Gignac et al., 2016; Gignac & Kley, 2014; Holliday et al., 2022). Crucially, this has enabled dissection of rare and recently extinct specimens from museum collections, as diffusible iodine staining is largely reversible (Early et al., 2020; Hedrick et al., 2018; Lanzetti & Ekdale, 2021; Leonard et al., 2022; Yapuncich et al., 2019). This technique has also facilitated the study of minute anatomical structures that are not possible to extract using traditional techniques (e.g. bird cranial and pectoral muscles: Jones et al., 2019; To et al., 2021; Widrig et al., 2023). Additionally, diceCT data have been used to create 3D interactive models (Bribiesca‐Contreras & Sellers, 2017; Holliday et al., 2013; Lautenschlager et al., 2014; Tsai & Holliday, 2011), which can be used for educational materials and further research (Gray et al., 2023). For example, subsequent biomechanical models have been created to investigate the impact of different morphologies on mechanical performance, for example reptile limb motion (Demuth et al., 2020; Tsai et al., 2020; Wilken et al., 2019) and feeding mechanics in rodents (Cox & Faulkes, 2014), frogs (Kleinteich & Gorb, 2015), primates (Orsbon et al., 2020) and bats (Santana, 2018). Even studies of extinct taxa have benefited from diceCT through increased contrast of internal fossil structures (Bailleul et al., 2021) and reconstructions of soft tissues supplemented with inferences of bony correlates (Lautenschlager, 2016). However, there are relatively few comparative studies incorporating enough diceCT data to analyse the relationship between soft‐tissue anatomy, ecology, phylogeny and behaviour across more than just a handful of species. Studies include the investigation of hindfoot drumming of mole‐rats (Sahd et al., 2022), bat diet (Santana, 2018) and flight performance (Stanchak & Santana, 2018), and masticatory mechanics in rodents (Hautier et al., 2012). For frogs, diceCT has only recently been applied to exploring frog anatomy (Collings & Richards, 2019; Porro & Richards, 2017). Only one study has used diceCT to compare muscle anatomy across taxa in relation locomotor behaviours, which was specifically in relation to the role of the forelimbs and pectoral girdle in determining burrowing type for five species (Keeffe & Blackburn, 2020). The novel combination of the resulting 3D anatomical reconstructions with biomechanical modelling of frog locomotion is rarer still (Collings et al., 2022).

The overarching aim of this paper is to quantify the size of the muscles in the pelvis and hindlimb using digital dissection and compare them across all five primary locomotor modes spanning all major phylogenetic groups. Based on the findings of previous literature outlined above, we hypothesise that: (1) there is a positive correlation between the length of each long bone in the pelvis and hindlimb and the size of their associated muscles; (2) there is a positive correlation between the length of the dorsal iliac and urostylic crests and the size of their associated muscles; (3) the distribution of muscle mass among the pelvis and hindlimb segments is a predictor of locomotor mode; and (4) differences in muscle separation between species observed in previous literature (Dunlap, 1960; Porro & Richards, 2017; Přikryl et al., 2009) could be explained by variation in locomotor mode (4.1) or by variation in phylogenetic position (4.2).

## 2 METHODS

### 2.1 Dataset

All specimen information and scan sources, staining durations and scanning parameters can be found in the Supplementary Dataset (DOI: 10.6084/m9.figshare.26357395). Information on locomotor mode was gathered from the literature (e.g. Jorgensen & Reilly, 2013; Keeffe & Blackburn, 2020) and through exchanges with researchers who have conducted first‐hand behavioural observations in the field (Andrew Gray and David Blackburn, pers. comms.). We use 30 taxa in this study, six from each locomotor mode—swimmers (AQ), walker‐hoppers (WH), burrower‐walker‐hoppers (BWH), terrestrial jumpers (TJ) and arboreal jumpers (AJ) (Wells, 2007). In line with previous literature, jumpers are differentiated from WH as frogs which can perform a leap greater than eight times their snout‐vent length and choose to jump more often than they walk (Emerson, 1979; Reilly et al., 2015; Soliz et al., 2017). We also decided to examine a mix of forward (*Hemisus guineensis*, *Rhinophrynus dorsalis*), backward (*Breviceps poweri*, *Neobatrachus pictus*) and non‐descript (*Bufo bufo*, *Phrynomantis annectans*) burrowers. Most μCT scans were obtained from MorphoSource.org and previous literature (Collings & Richards, 2019; Porro & Richards, 2017). To complete the coverage of locomotor modes, two additional specimens were stained and μCT‐scanned (see Section 2.3).

### 2.2 Phylogeny

To determine the importance of phylogenetic history in the evolution of skeletal structures, Jetz and Pyron's (2018) phylogeny was trimmed down to the taxa used in the present study using the ‘keep. tip’ function in *ape* (Paradis & Schliep, 2019; R Version 4.3.1, 2020). The phylogeny was updated with the most recent nomenclature according to the IUCN (2023). This phylogeny was used to allocate each species to broad phylogenetic groups for the PERMANOVA (see Section 2.8)—Archaeobatrachia, Ranoidea or Hyloidea. *Neobatrachus pictus* and *Sechellophryne gardineri* are sister taxa of Hyloidea and Ranoidea, respectively (Portik et al., 2023), so they are referred to simply as Neobatrachia for this part of our study.

### 2.3 Specimen preparation and staining

Lugol's iodine enables visualisation of soft tissues which would otherwise be indistinguishable from each other by increasing their radiopacity (Gignac et al., 2016; Metscher, 2009). The iodine selectively binds to the glycogen molecules and lipids within the muscles (Li et al., 2016). This process has been shown to cause varied levels of muscle shrinkage during staining at high concentrations (>10%) and long durations (Hedrick et al., 2018; Lanzetti & Ekdale, 2021; Vickerton et al., 2013). The nine scans performed prior to the publication of Hedrick et al. (2018) were stained using iodine concentrations of 7.5% or less, with most scans we use being of specimens stained with 1.25% Lugol's iodine (Supplementary Dataset). The mechanism of shrinkage has only recently been diagnosed as being caused by the acidification of the iodine (Dawood et al., 2021). Incorporating a buffer into the Lugol's solution stabilises the pH and significantly reduces shrinkage due to staining while preserving the high‐resolution contrast (Gray et al., 2023). Therefore, the final two specimens we added to our dataset were stained using 1.25% buffered Lugol's (Supplementary Dataset).

Where possible, any museum specimens were chosen from containers with many individuals from the same locality, avoiding frogs that were relatively small (i.e. potentially juveniles), had signs of damage (i.e. broken bones and previous physical dissection) or had any limb bent into unnatural positions. All specimens chosen appeared to have a ‘relaxed’ or ‘natural’ pose to avoid overestimating muscle lengths from overly stretched muscles. As specimens had been fixed in formalin and stored in ethanol, and the staining solution is water‐based, specimens were placed in a new glass jar containing 50% ethanol, then the solution was replaced with 30% ethanol after a couple of days (Gray et al., 2023). After a total of 1 week, specimens were stained in jars of buffered Lugol's iodine. Staining time varied depending on the size of the specimen, but as most frogs are relatively small, it was around 1–2 weeks (Supplementary Dataset). After the final scan was complete, specimens were de‐stained using step‐wise concentrations of fresh ethanol (Gray et al., 2023). Staining is not entirely reversible as soft tissues can remain more radiopaque than before (Figure S2; Early et al., 2020) but this method removes the physical stain to a sufficient level to return to the museum collection.

### 2.4 μCT scanning

Specimens were scanned prior to staining to better visualise the skeletal structures, as they can be more difficult to segment after the contrast of soft tissues has been enhanced. An X‐ray filter was used if the specimen was particularly large or dense (Supplementary Dataset). For stained specimens, the iodine solution can accumulate at the boundary between the skin and the muscles due to differences in binding abilities and rates of transport between types of soft tissue, decreasing scan quality (Li et al., 2016). Therefore, specimens were submerged in a water bath at room‐temperature for at least half an hour prior to scanning to remove unbound iodine. Most scans were conducted as a series of overlapping ‘panels’ along the same vertical axis to achieve high (<20 μm/voxel) resolution for each region of interest (Supplementary Dataset).

### 2.5 Digital dissection

**FIGURE 1:**
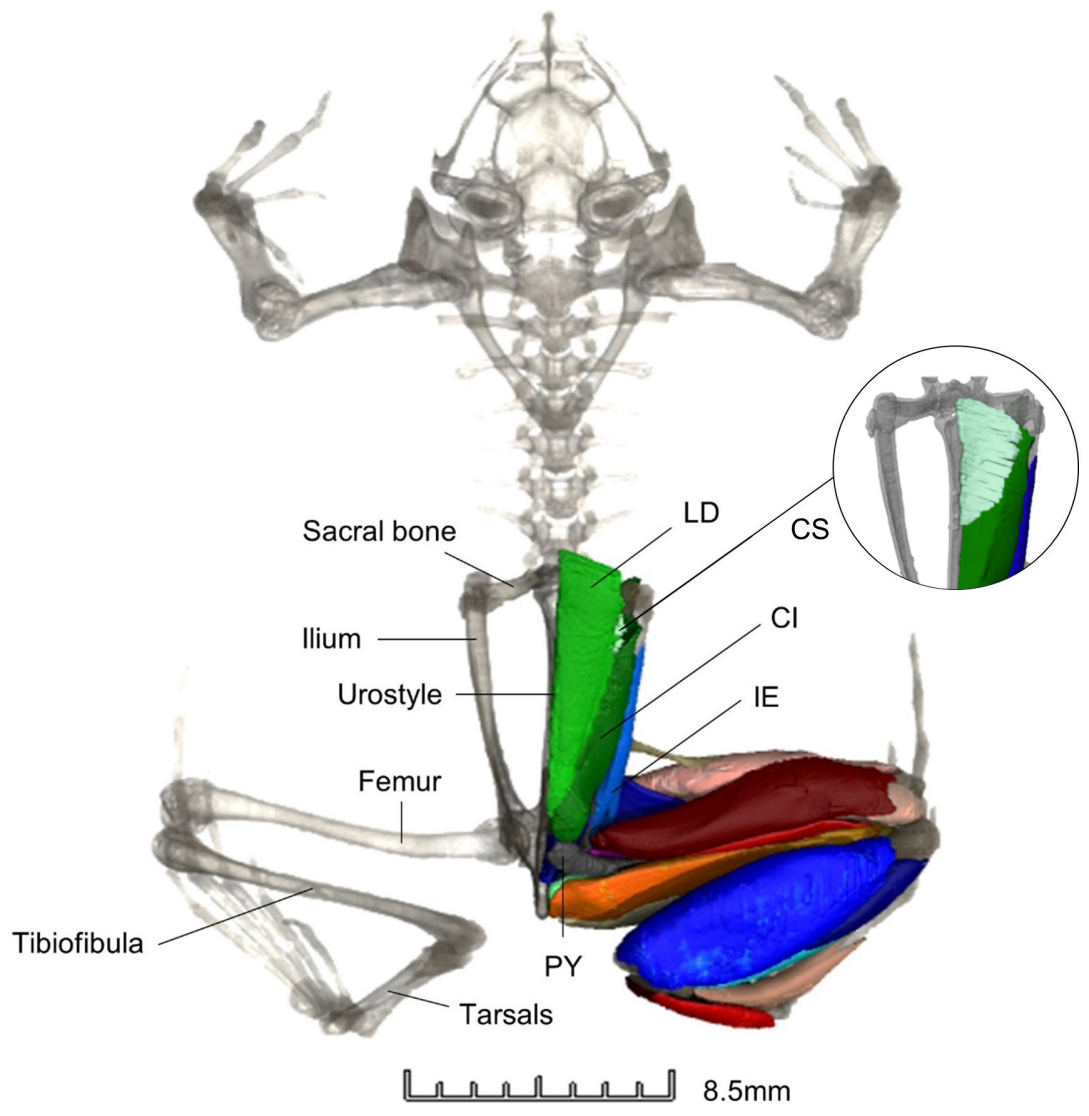
3D digital dissection of *Hemisus guineensis* (voucher number: CAS‐herp‐258533) in VGStudio Max (Version 3.4), with annotation of the skeleton and pelvic muscles in dorsal view. The view of the coccygeosacralis (CS) is often obscured as it is positioned behind the longissimus dorsi (LD). Coccygeoiliacus (CI); iliacus externus (IE); pyriformis (PY).

**FIGURE 2:**
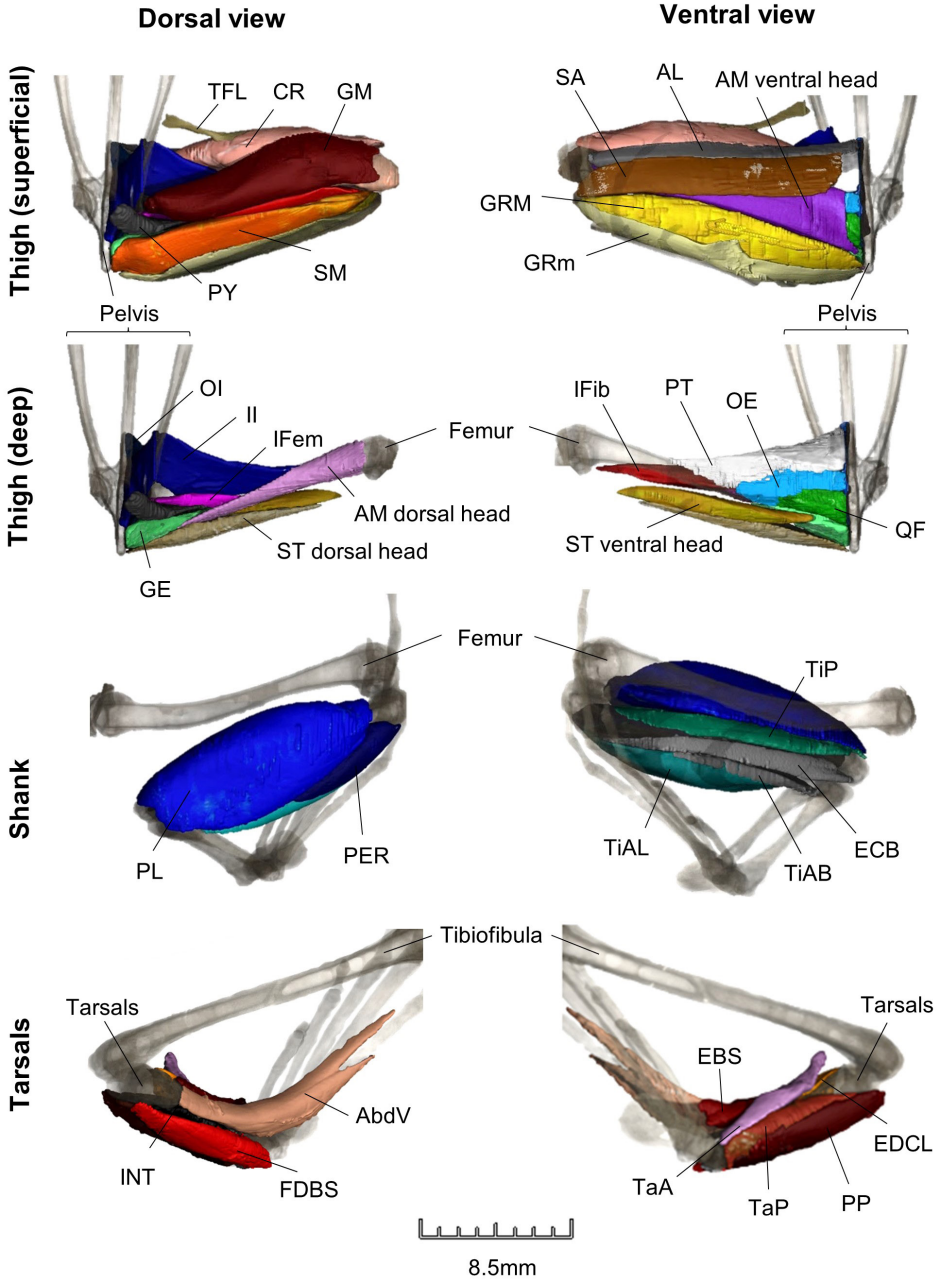
3D digital dissection of the right hindlimb of *Hemisus guineensis* (voucher number: CAS‐herp‐258533) in VGStudio Max (Version 3.4). See Table 1 for the full names of each thigh and shank muscle. Proximal foot muscles: adductor brevis dorsalis V (AbdV); extensor brevis superhallucis (EBS); extensor digitorum communis longus (EDCL); flexor digitorum brevis superficialis (FDBS); Intertarsalis (INT); plantaris profundus (PP); tarsalis anticus (TaA); tarsalis posticus (TaP).

Thirteen scans had been digitally dissected for previous studies (e.g. Collings & Richards, 2019; Porro & Richards, 2017) in Avizo (Version 8.0). Eight scans were digitally dissected in Amira (Version 2020.2), while the remaining nine scans were segmented in VGStudio Max (Version 3.4). Digital dissection was carried out from the pelvis (excluding the iliolumbaris, as it extends from the sacral bone anteriorly) to the proximal foot (excluding small foot muscles that originate at the tarsometatarsal joint) for whichever hindlimb showed the least damage (Figures 1 and 2). Muscle topology, variation in muscle fibre orientation and differences in density between tissues were used to discriminate between structures. Muscle nomenclature and abbreviations are consistent with previous literature on frog dissection (Collings & Richards, 2019; Dunlap, 1960; Porro & Richards, 2017; Přikryl et al., 2009). In Avizo/Amira, the threshold tool in the segmentation editor was used to isolate bone and soft tissue. Individual structures were selected using the paintbrush tool no more than every five slices before using the interpolation tool. In VGStudio Max, a combination of the draw, region growing, opening/closing, erode/dilate, smoothing and refinement tools were used. This programme interpolates the changes across all three planes of view and updates the 3D rendering automatically.

Separate meshes for each of the anatomical components were then generated in Amira/VGStudio and exported to Blender (Version 3.5), where the meshes were smoothed and animated to facilitate the visualisation of deeper muscles. The finished 3D models were then annotated and made freely available for download in Sketchfab (https://sketchfab.com/aleavey/collections) for use in future research and as educational materials.

### 2.6 Extracting gross anatomical muscle data

**FIGURE 3:**
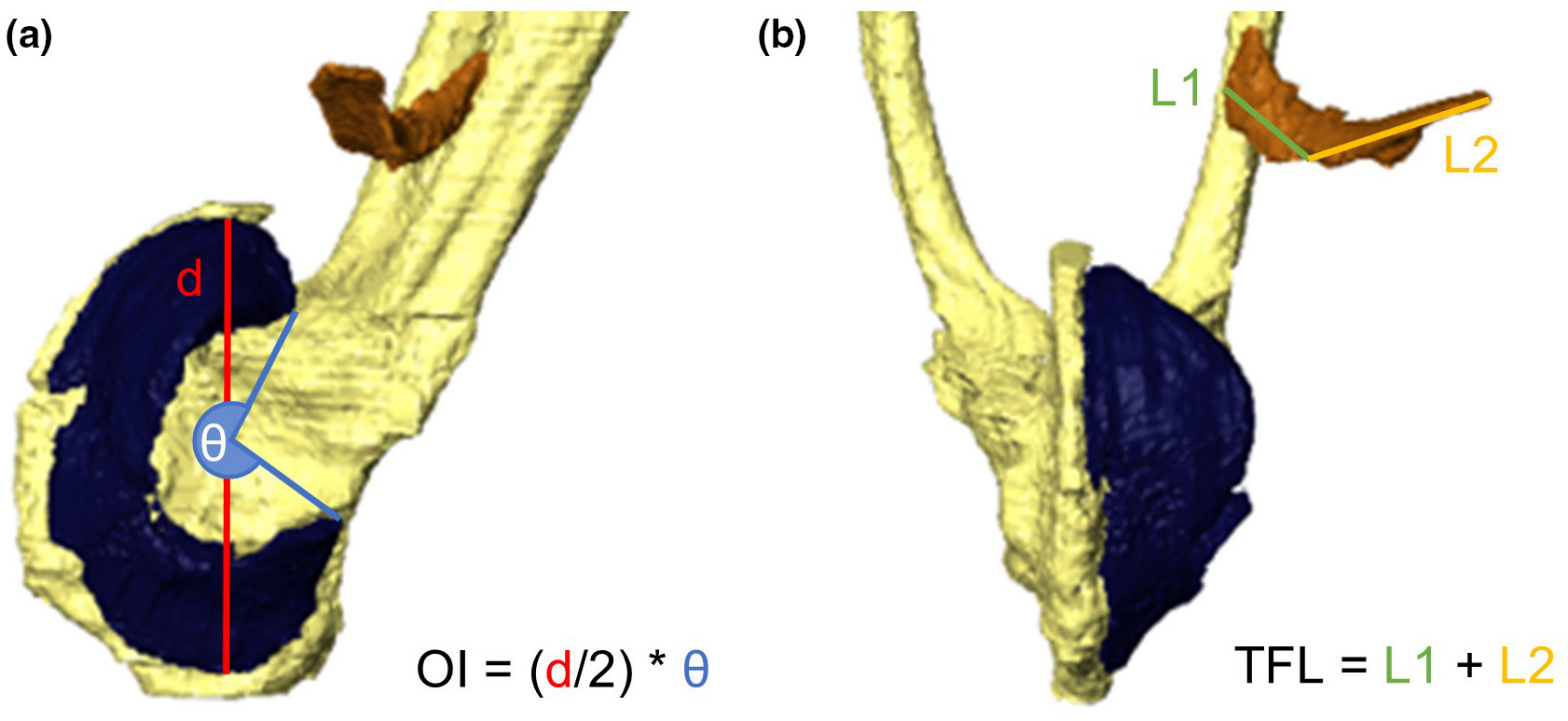
Techniques for measuring the length of curved muscles (a) for obturator internus (OI) (side view) and (b) all other curved muscles, using tensor fascia latae (TFL) as an example (ventral view), shown in Amira (Version 2020.2) using *Phlyctimantis maculata* (specimen from Porro & Richards, 2017). The longest possible distance between the proximal and distal end of these curved muscles (dashed line) is used to determine the two points to measure from.

Snout‐vent length (SVL), the length of each hindlimb segment and the length of the dorsal crests on the ilium and urostyle were recorded using a 3D line tool. Muscle belly length (MBL), defined as the longest distance between the proximal origin and distal insertion point of each muscle (Lieber & Fridén, 2000), was also measured for each muscle in the pelvis and hindlimbs. The longissimus can originate as far anteriorly as the pectoral girdle in some species (Přikryl et al., 2009), but this was often too difficult to dissect completely due to the presence of many layers of muscle divided by transverse tendinous septa. Therefore, the longissimus was measured from its point of attachment on the anterior side of the sacral bone to its point of insertion on the urostyle since this area of anatomy was most important for addressing the aims of this paper. Most curved muscles were measured using the sum of two parts to reduce the chances of measurement error—a straight‐line measurement from each end of the muscle, meeting on the outer edge of the centre of the curve (Figure 3). Obturator internus (OI) originates from the ischium and wraps around the proximal head of the femur. The length of OI was calculated by multiplying the radius and central angle to obtain more replicable results across scans compared with a series of short straight‐line measurements (Figure 3). Muscle belly volume was extracted from the segmentation software and multiplied by the standard value for vertebrate skeletal muscle density (1.056 g/cm^3^; Mendez & Keys, 1960) to calculate muscle belly mass (MBM), a measure for the muscle's inertial resistance against translation (Nauwelaerts et al., 2007).

To address Hypothesis 4, the total number of muscle heads was counted for the pelvis, thigh and shank. Separate muscle heads were defined as when there is a distinct and consistent area of lower grayscale values between two (or more) areas of muscle that could be traced in all planes of view. A muscle head was considered separate if this occurred throughout at least one third of the length of the muscle, as there could be variation in muscle function even when there is separation at only one end of the muscle (Collings & Richards, 2019). Where possible, the literature was consulted to check if muscle separation had been found during traditional dissection (Collings & Richards, 2019; Porro & Richards, 2017; Přikryl et al., 2009). The semimembranosus and gracilis major thigh muscles were not considered reliable to assess for this step as they are known to have oblique tendinous inscriptions (Collings & Richards, 2019; Přikryl et al., 2009), and tendons cannot be visualised in iodine‐stained scans. These two muscles, and all the muscles where the belly appeared entirely whole, were counted as one head. The proximal foot was excluded from the muscle head analysis because the distal part of the hindlimb is where scan resolution tended to be lowest, making the ability to distinguish between different heads too difficult to be reliable.

### 2.7 Considering potential variation in muscle shrinkage and body size

Specimens stored in alcohol‐based solutions are more likely to exhibit muscle shrinkage than those which are scanned after being thawed from frozen (Leonard et al., 2022). Higher concentrations of iodine, staining for longer durations, and/or using Lugol's without a buffer also increase the extent of muscle shrinkage (Dawood et al., 2021; Vickerton et al., 2013). The specimens used in the present study differed in the duration of storage, the type of solution they were stored in, and the concentration and duration of staining, meaning that there is variation in the mass and density of muscles (Levy, 2018). This variation will have consequences for the inferences of functional capabilities of the muscles in each specimen. Additionally, total body mass is not readily available for most of the specimens for shrinkage corrections suggested by previous studies (Leonard et al., 2022). Therefore, the mass of each muscle was converted into relative percentages of the total muscle mass of each segment. The length of each bone and muscle was considered relative to snout‐vent length (SVL). All continuous data (excluding muscle head number, see Section 2.8) were log‐transformed (log+1) prior to any statistical analyses. Using this approach also reduces the effect of sexual dimorphism, as a mix of sexes had to be used and females are larger than males in approximately 90% of frog species (Nali et al., 2014).

### 2.8 Statistical analyses

All analyses were carried out in R (Version 4.3.1). The residuals for all variables were tested to see if they have a normal distribution while controlling for phylogeny using Shapiro–Wilk tests (Revell, 2009; Table S1). The phylogenetic signal of each variable was then estimated using the ‘phylosig’ function in *phytools* (Revell, 2012), and then, the ‘fitContinuous’ function was used to determine the best model of evolution for subsequent analyses. Brownian motion was the best model for 65.4% of variables and second‐best in 26.9% of models (Table S2), so a covariance matrix based on a Brownian motion model of evolution (‘vcv.phylo’ function in *ape*) was used in all subsequent analyses where phylogenetic relatedness is incorporated.

Tests for differences between locomotor modes were most often standard ANOVAs followed by post hoc Tukey tests, or Kruskal–Wallis tests followed by Dunn's tests if the residuals are not normally distributed, since the majority of variables show no phylogenetic signal (Table S2). Where a variable showed evidence of phylogenetic signal, a phylogenetic analysis of variance (phylANOVA) or, if the residuals are not normally distributed, a Residual Randomization in Permutation Procedure (RRPP) prior to running the phylANOVA (Collyer & Adams, 2018) was used. A Bonferroni correction was integrated into all pairwise comparisons to correct for multiple testing. Any tests between multiple continuous variables (e.g. bone length and muscle mass/length) were performed using an ordinary least squares (OLS) regression and a phylogenetic generalised least squares (PGLS) analysis in the *caper* package (Orme et al., 2013).

To address Hypothesis 1, we tested for correlations between bone length and the mass and length of each associated muscle. The pyriformis, tensor fascia latae and obturator internus were excluded from this stage of analysis as they do not run parallel to any long bones. To address Hypothesis 2, differences between locomotor modes in the length of the dorsal crests on the ilia and urostyle were examined, and regression models were used to determine whether there is a correlation between dorsal crest length and the mass of any muscles which attach to these crests. To address Hypothesis 3, the relative masses of each thigh and shank muscle were added together according to the functional muscle groups described in the literature (Table 1). Additionally, we calculated the total muscle mass within the pelvis and each hindlimb segment relative to the total muscle mass across all segments to get an estimation of how muscle is distributed across post‐vertebral anatomy. We then analysed the differences between locomotor modes in total relative muscle mass for the pelvis, each hindlimb segment and each muscle functional group.

**TABLE 1:** Summary of the thigh and shank muscles which have similar functions according to Přikryl et al. (2009) and Duellman and Trueb (1986).

Functional group	Muscles
Thigh	
Femur retraction	Semimembranosus (SM), iliofibularis (IFib), gemellus (GE), obturator externus (OE), quadratus femoris (QF)
Femur protraction and adduction	Adductor magnus (AM), sartorius (SA), adductor longus (AL)
Femur retraction and adduction	Gracilis major (GRM), iliofemoralis (IFem), gracilis minor (GRm)
Femur protraction and abduction	Iliacus internus (II)
Femur long‐axis rotation	Obturator internus (OI)
Femur stabilisation (i.e. resistance to long‐axis rotation)	Pectineus (PT)
Knee flexion	Semitendinosus (ST)
Knee extension	Cruralis (CR), gluteus magnus (GM), tensor fascia latae (TFL)
Shank	
Ankle extension	Plantaris longus (PL), tibialis anticus longus (TiAL), tibialis posticus (TiP)
Knee extension	Peroneus (PER), extensor cruris brevis (ECB)
Dorsiflexion and inversion of the ankle	Tibialis anticus brevis (TiAB)

Four phylogenetic principal component analyses (pPCAs), one for the pelvis and one for each hindlimb segment, were performed using relative muscle masses to find the principal axes of variation in the muscle composition within each area of post‐vertebral anatomy. These analyses were carried out under a Brownian motion model of evolution on the covariance matrix (phyl.pca function in *phytools*; Revell, 2012). The first two pPCs from each analysis were plotted to examine how species cluster according to locomotor mode and phylogenetic group (Archaeobatrachia, Neobatrachia, Hyloidea and Ranoidea). A permutational multivariate analysis of variance (PERMANOVA) was then used to test whether the differences between group means for each locomotor mode and phylogenetic group was significant by performing pairwise comparisons (*pairwiseAdonis* package; Anderson, 2005). All PERMANOVAs used 999 permutations and corrected for multiple testing by adjusting the *p*‐values using a Bonferroni correction.

The total number of muscle heads in each area of post‐vertebral anatomy was treated as continuous (but left un‐transformed), rather than discrete data, because partial fusion/separation was observed at the proximal or distal ends of muscles in some species. This approach preserves the order of the data, as well as the upper and lower bounds (e.g. you cannot have less than 17 and more than 23 thigh muscle heads). To address Hypothesis 4.1, an ordinary least squares (OLS) regression was fit to pelvic, thigh and shank muscle head numbers to evaluate the differences between locomotor modes without the incorporation of phylogenetic relatedness. The results were compared with an identical set of PGLS models to address Hypothesis 4.2, that is to incorporate phylogenetic history as a potential explanatory factor. Voxel size was also included as a random factor in the muscle head number and total muscle mass distribution analyses to test whether a low resolution relative to actual structure size in smaller specimens might cause over‐estimations in object size (Broeckhoven & du Plessis, 2018).

## 3 RESULTS

### 3.1 Pelvic muscle anatomy

Regarding their relative length, all pelvic muscles excluding the pyriformis show some evidence of a phylogenetic signal (Table S2). In support of Hypothesis 1, the length of the iliac externus muscle is positively correlated with the length of the ilium, while coccygeoiliacus length is positively correlated with urostyle length (Table 3). There are no significant differences between locomotor modes in the length of the pelvic bones or muscles (Table S3). General observations include the longissimus dorsi inserting further posteriorly along the length of the urostyle on average in non‐jumpers, WH having a slightly longer coccygeosacralis relative to urostyle length, the coccygeoiliacus being considerably shorter in AJ than all other locomotor modes, and both swimmers and jumpers having a relatively longer iliacus externus than other locomotor modes (Figure S3).

**FIGURE 4:**
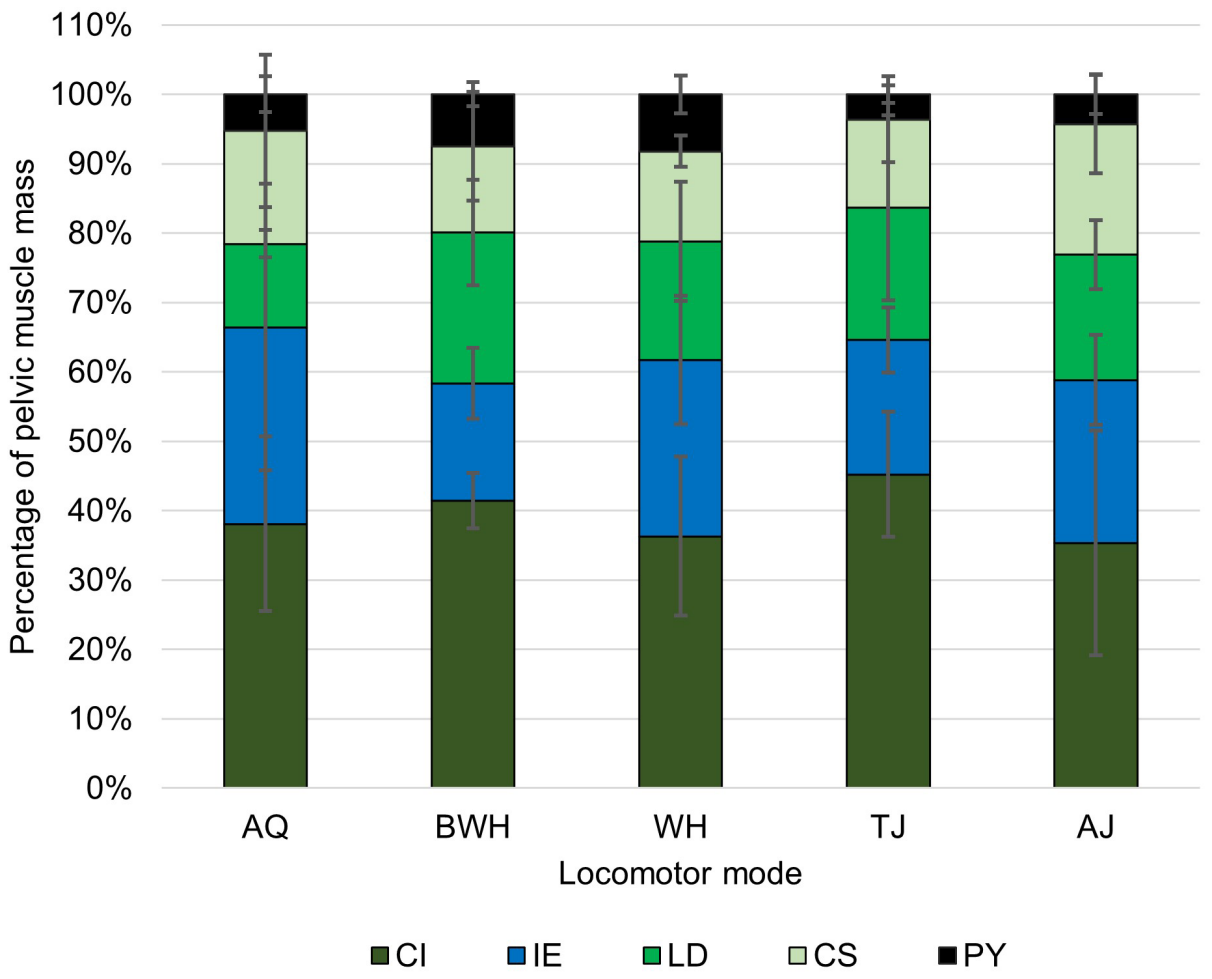
Average mass across locomotor modes for muscles in the pelvis prior to log transformation. The error bars represent standard deviation from the mean. There are no pairwise significant differences between locomotor modes. See Figure 1 for the full names of anatomical abbreviations.

**FIGURE 5:**
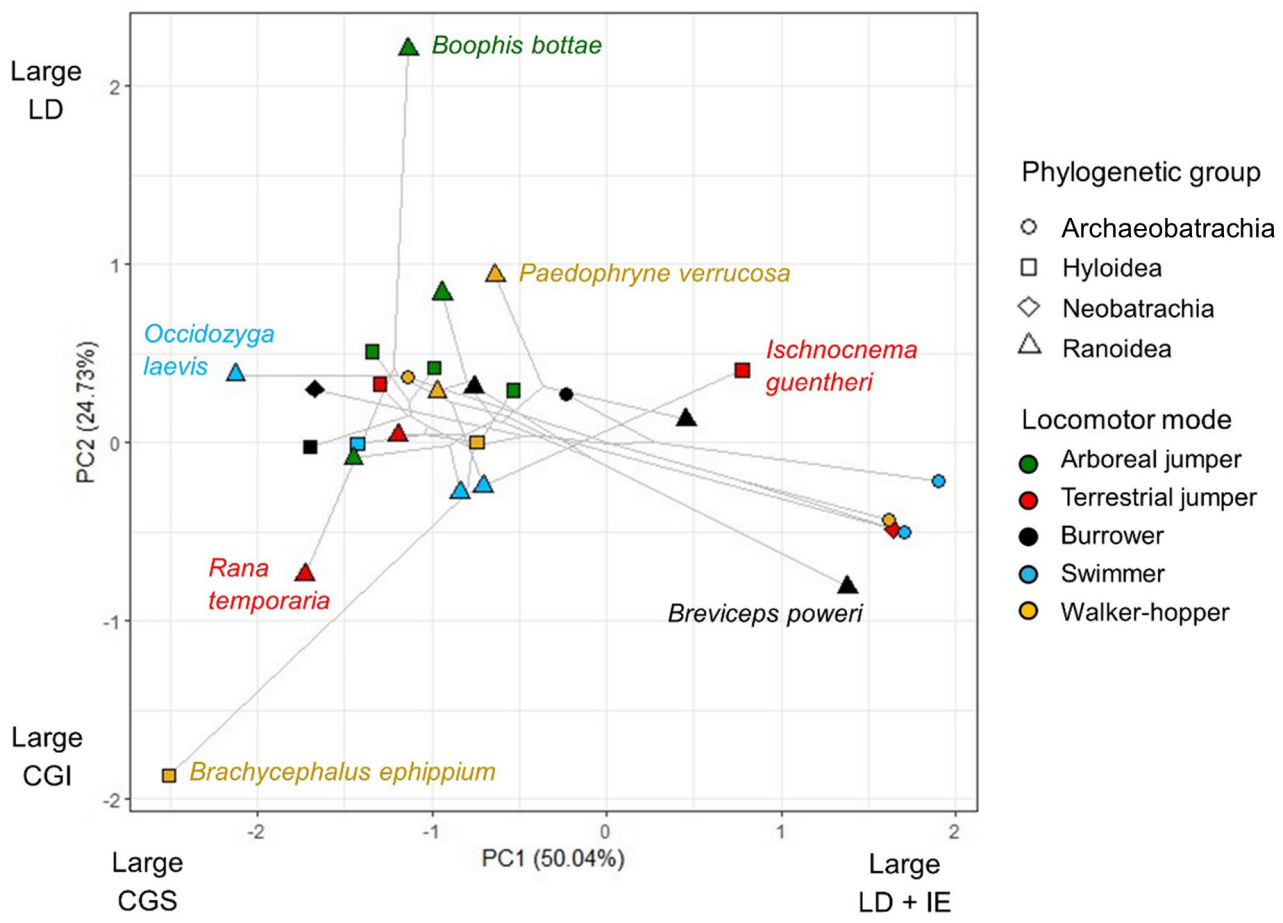
A plot of the first two phylogenetic principal component (PC) values generated from the relative masses of the pelvic muscles, coded by phylogenetic group and locomotor mode. Species with noticeably different morphologies have been labelled. Axes are labelled with the muscles most strongly influencing the positive and negative loadings (Table S4). CI, coccygeoiliacus; CS, coccygeosacralis; IE, iliacus externus; LD, longissimus dorsi; PY, pyriformis.

Regarding the relative mass of each pelvic muscle, the coccygeosacralis is the only muscle with evidence of a phylogenetic signal (Table S2). None of the muscles show any correlation between mass and the length of its associated pelvic bone, in contrast to Hypothesis 1. The pyriformis is the only muscle to show a significant difference in mass between locomotor modes (ANOVA: *F*_(4,25)_ = 3.24, *p* = 0.028), though there are no significant pairwise differences (Figure 4). According to the phylogenetic PCA, variation in pelvic muscle composition is primarily driven by the relative mass of the longissimus dorsi, iliacus externus and coccygeosacralis in pPC1, and the longissimus dorsi and coccygeoiliacus in pPC2 (Figure 5; Table S4). The first three pPCs attained 95.9% accumulative variance. The error bars in Figure 4 and spread of taxa across Figure 5 show that WH have the largest variation in pelvic muscle anatomy, while AJ have the smallest. There are no significant differences in the area occupied by each locomotor mode along either axis, but there is a significant difference between the Archaeobatrachia and both the Hyloidea and Ranoidea along pPC1 (Table 2), where Archaeobatrachia tend to have a larger iliacus externus (Figure 5).

**TABLE 2:** Summary of the pairs of locomotor modes and phylogenetic groups which differ significantly in muscle anatomy according to PERMANOVA tests of the phylogenetic principal components (PC) for each hindlimb segment.

Model	Pairs	*F* model	*R* ^2^	*p‐*value	Adjusted *p‐*value
Pelvis PC1	**Archaeobatrachia vs. Hyloidea**	9.419	0.44	0.007	**0.042**
	Archaeobatrachia vs. Ranoidea	8.457	0.346	0.009	0.054
Pelvis PC2	AJ vs. WH	3.452	0.257	0.049	0.49
	AJ vs. AQ	3.275	0.247	0.049	0.49
Thigh PC1	**TJ vs. BWH**	20.86	0.676	0.002	**0.02**
	AJ vs. TJ	10.768	0.519	0.012	0.12
Thigh PC2	AQ vs. BWH	8.172	0.45	0.028	0.28
Shank PC1	BWH vs. AJ	17.286	0.634	0.008	0.08
	BWH vs. AQ	9.421	0.485	0.016	0.16
	BWH vs. TJ	9.336	0.483	0.007	0.07
Shank PC2	AJ vs. TJ	13.237	0.57	0.006	0.06
	AJ vs. AQ	12.747	0.56	0.011	0.11
	BWH vs. AQ	5.755	0.365	0.04	0.4
PF PC1	AQ vs. AJ	9.088	0.476	0.014	0.14
	AQ vs. WH	8.294	0.453	0.013	0.13
	TJ vs. AJ	7.744	0.436	0.028	0.28
	TJ vs. WH	6.923	0.409	0.028	0.28

*Note*: The first locomotor mode described in the pairing has the higher PC value. Pairs highlighted in bold are significantly different even after the *p‐*value has been adjusted for multiple testing using a Bonferroni correction.

Abbreviation: PF, proximal foot.

The lengths of the dorsal crests on the ilia and urostyle show evidence of a phylogenetic signal, where crests are present and longer in more recently evolved taxa (Table S2). However, neither the ilia (phylANOVA: *F* = 1.24, *p =* 0.275) nor urostyle (phylANOVA: *F* = 0.59, *p =* 0.617) show significant differences in crest length between locomotor modes. Interestingly, muscle mass increases significantly with the length of the attachment sites on the iliac shaft and/or urostyle (i.e. excluding the crest, if present) for all muscles except the iliacus externus, but only coccygeosacralis mass increases significantly with the length of the attachment site on the urostylic crest, suggesting only partial support for Hypothesis 2 (Table S5). Additionally, there are no significant correlations between crest lengths and the length of any of the pelvic muscles.

### 3.2 The total muscle mass of each hindlimb segment

**FIGURE 6:**
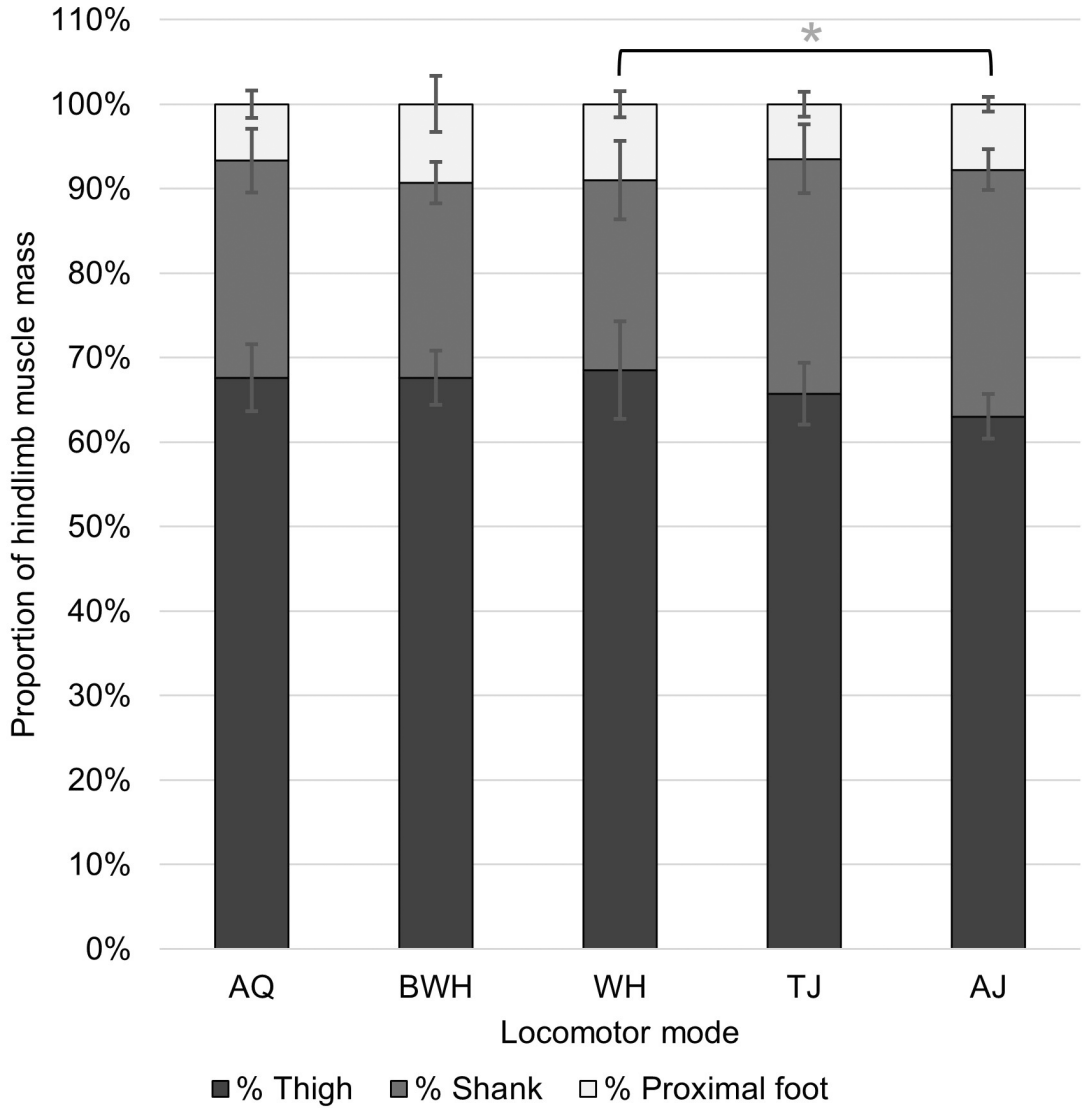
Average muscle mass in each hindlimb segment across locomotor modes. The error bars represent standard deviation from the mean. The Tukey test significance values refer to the log‐transformed variables and are represented by *p < 0.05.

Based on the standard deviation of total relative muscle mass across all taxa, differences in muscle distribution across the hindlimb segments are driven mostly by the thigh (4.44%) and shank (4.43%), while the proximal foot is relatively uniform (2.26%). None of the variables describing the total relative muscle mass in each hindlimb segment show any phylogenetic signal, but the relative length of the calcaneus does (Table S2). The total relative muscle mass of the shank is positively and significantly correlated to its length relative to SVL (OLS: *t* = 3.10, *p* = 0.004), but this relationship is not significant for the thigh (OLS: *t* = −1.31, *p* = 0.201) and proximal foot (PGLS: *t* = −0.99, *p* = 0.332). In terms of differences between locomotor modes, WH, AJ and BWH have the highest total thigh, shank and proximal foot muscle masses, respectively (Figure 6). WH have the most variable hindlimb muscle distribution, while AJ deviates least from the average. The shank is the only segment which shows any significant differences in total muscle mass between locomotor modes (ANOVA: *F*_(4,25)_ = 3.15, *p* = 0.032). The only locomotor modes which differ significantly from each other are AJ and WH, where AJ have a higher relative shank muscle mass (Tukey: difference in means = 6.71%, *p* = 0.045; Figure 6). Adding voxel size as another model parameter does not improve the fit to the data, indicating that scan resolution does not significantly impact the findings (Table S6).

### 3.3 Muscle anatomy within each hindlimb segment

**FIGURE 7:**
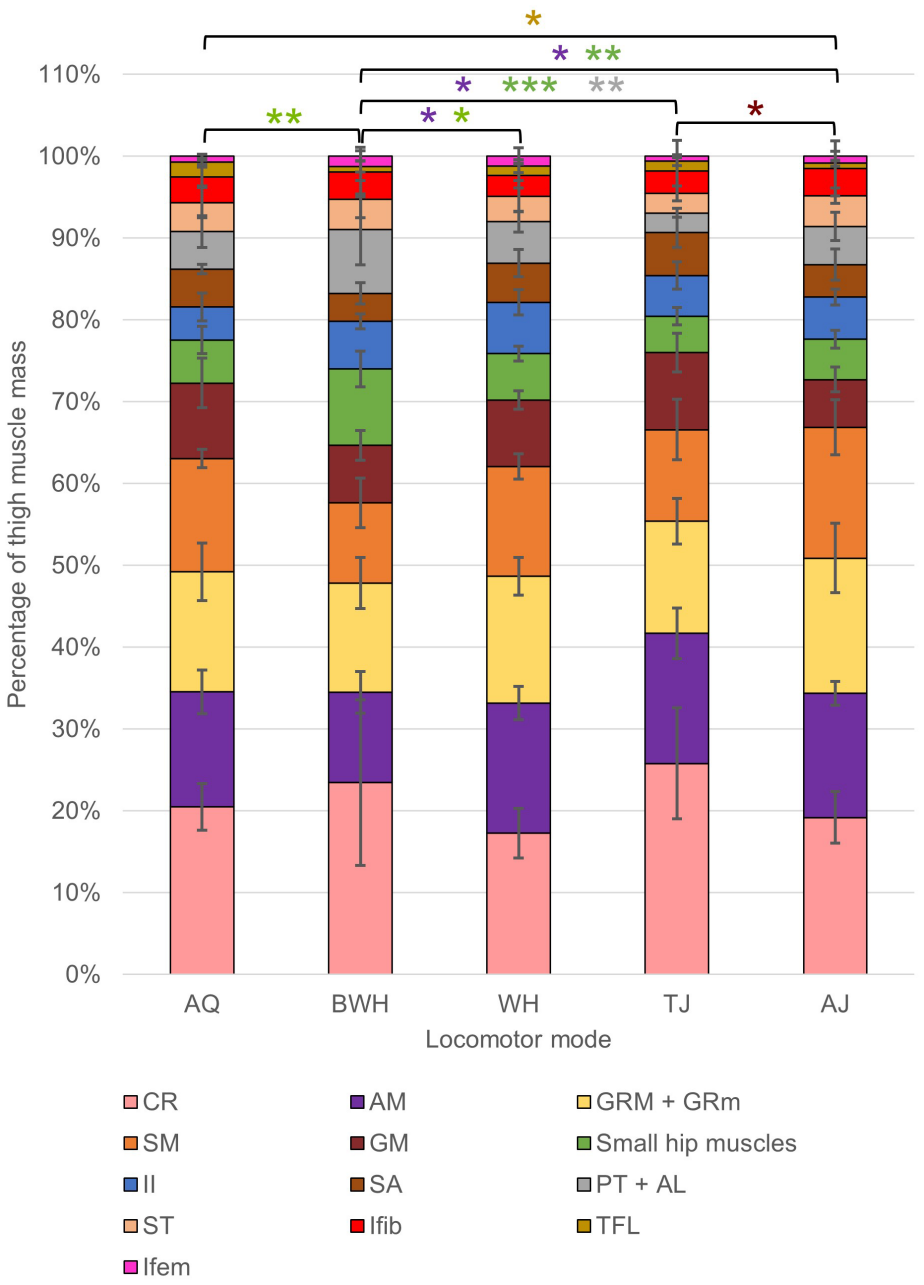
Average mass across locomotor modes for muscles in the thigh prior to log transformation. The error bars represent standard deviation from the mean. Pairwise phylANOVA (TFL and GM) and Tukey test significance values refer to the log‐transformed variables and are represented by ***p < 0.001, **p < 0.01 and *p < 0.05. See Table 1 for the full names of anatomical abbreviations.

**FIGURE 8:**
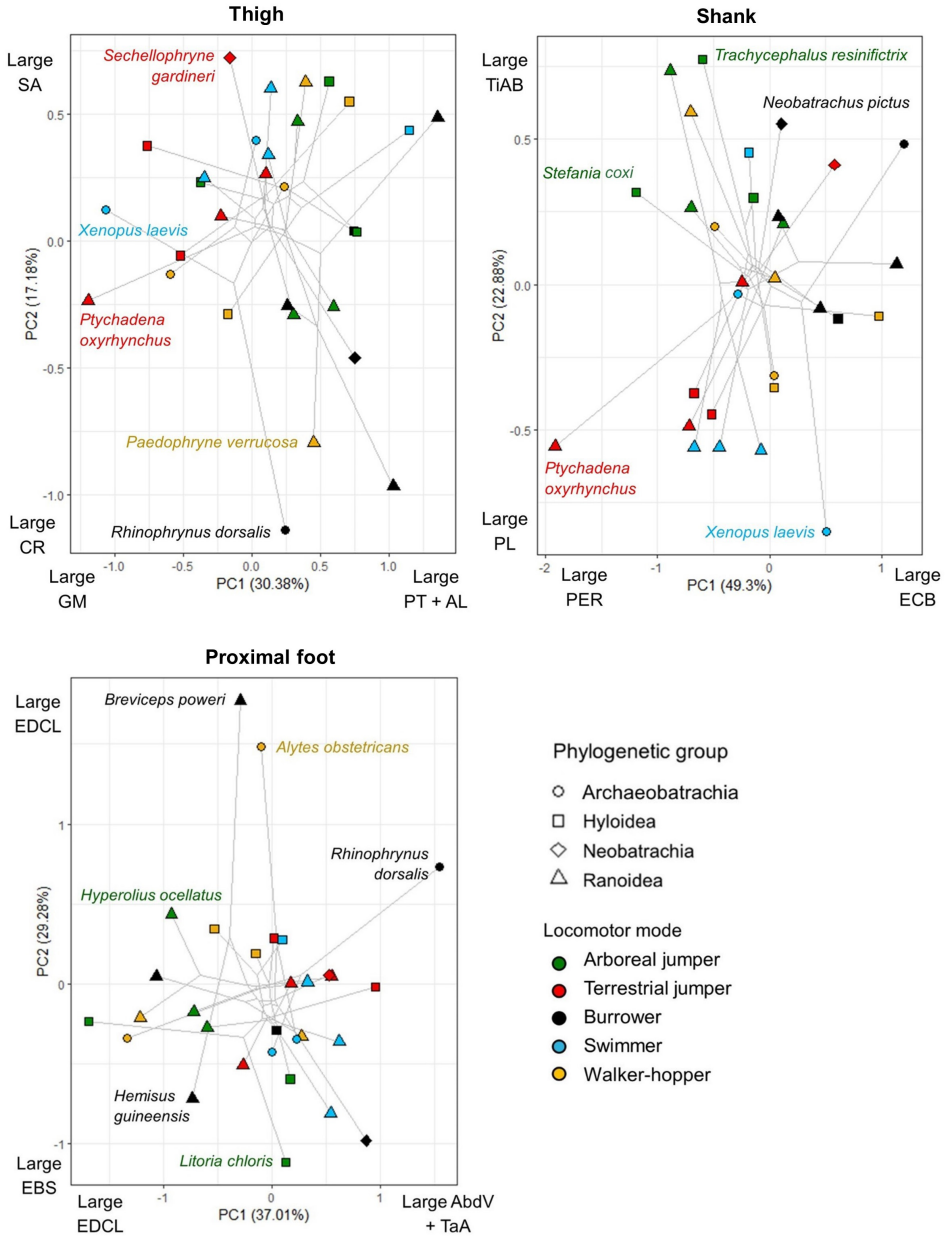
Plots of the first two phylogenetic principal component (PC) values generated from the relative muscle masses within each hindlimb segment, coded by phylogenetic group and locomotor mode. Species with noticeably different morphologies have been labelled. Axes are labelled with the muscles most strongly influencing the positive and negative loadings (Table S4). Significant differences in PC values between locomotor modes can be found in Table 2. AbdV, adductor brevis dorsalis V; AL, adductor longus; CR, Cruralis; EBS, extensor brevis superhallucis; ECB, extensor cruris brevis; EDCL, extensor digitorum communis longus; GM, gluteus magnus; PER, Peroneus; PL, Plantaris longus; PT, Pectineus; SA, sartorius; TaA, tarsalis anticus; TiAB, Tibialis anticus brevis.

**FIGURE 9:**
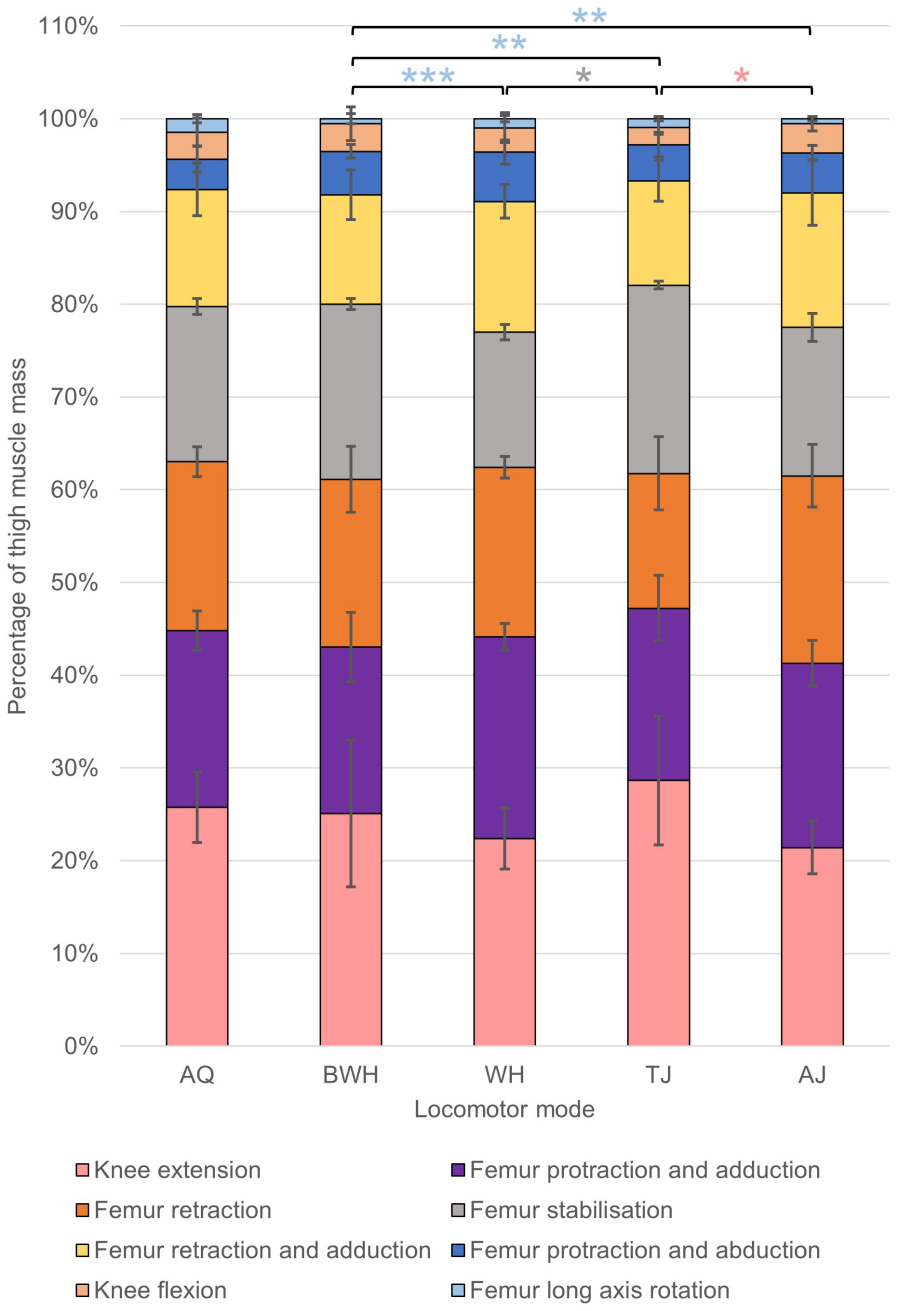
Average mass of each functional muscle group in the thigh prior to log transformation. The error bars represent standard deviation from the mean. See Table 1 for the muscles within each functional group. Colours match the muscle in Figure 7 that contributes most to each functional group. Pairwise phylANOVA (knee extension) and Tukey test significance values refer to the log‐transformed variables and are represented by ***p < 0.001, **p < 0.01 and *p < 0.05.

Regarding the thigh, many muscle lengths are positively correlated with femur length (Table 3), providing some support for Hypothesis 1, and show significant differences between locomotor modes (Table S3). However, only the adductor magnus shows a significant increase in mass with femur length (Table 3). Interestingly, the hip muscles and pectineus‐adductor longus complex decrease in mass with femur length. The muscle composition of the thigh (Figure 7) is primarily driven by variation in the relative mass of the pectineus, adductor longus and gluteus magnus along pPC1 and the sartorius and cruralis in pPC2 (Figure 8; Table S4). The first four pPCs explain 70.93% of the total variance, and BWH has the most variation in thigh musculature, while AQ has the least (Figures 8 and 9). TJ has a significantly smaller pPC1 value than BWH, which primarily reflects their larger gluteus magnus muscles (Table 2). For pPC2, AQ have higher values than BWH (Table 2), which reflects their larger sartorius muscle (Figures 8 and 9). There are no significant differences between phylogenetic groups along either axis. When thigh anatomy is evaluated in terms of functional muscle groups (Table 1), TJ showed the most deviation from group averages, while WH showed the least (Figure 9). None of the functional muscle groups in the thigh that show evidence of a phylogenetic signal (Table S2) differ significantly between locomotor modes. Femur stabilisers (ANOVA: *F*_(4,25)_ = 3.67, *p* = 0.017) and femur long‐axis rotators (ANOVA: *F*_(4,25)_ = 8.10, *p <* 0.001) show significant differences across locomotor modes. TJ have significantly larger femur stabilisers than WH, and the long‐axis rotators are significantly smaller in BWH compared with AJ, TJ and WH (Figure 9).

**TABLE 3:** Phylogenetic generalised least squares (PGLS) and ordinary least squares (OLS) regressions between the length of each long bone and the mass and length of each of its associated muscles.

	Model	*t*‐value	*p*‐value
PGLS	CI length ~ Urostyle length	2.053	0.0495
	IE length ~ Ilia length	2.16	0.0395
	AM (dorsal head) length ~ Femur length	5.151	<0.001
	ECB mass ~ Tibiofibula length	−4.332	<0.001
	FDBS mass ~ Calcaneus length	2.129	0.0422
	INT mass ~ Calcaneus length	−3.129	0.0041
	PP length ~ Calcaneus length	8.667	<0.001
	TaP length ~ Calcaneus length	9.839	<0.001
	TaA length ~ Calcaneus length	6.778	<0.001
	EDCL length ~ Calcaneus length	2.516	0.018
	FDBS length ~ Calcaneus length	10.527	<0.001
	INT length ~ Calcaneus length	8.72	<0.001
	AbdV length ~ Calcaneus length	3.902	<0.001
OLS	Small hip muscles mass ~ Femur length	−3.507	0.0016
	AM mass ~ Femur length	2.145	0.0408
	PT + AL mass ~ Femur length	−3.757	<0.001
	CR length ~ Femur length	10.138	<0.001
	GM length ~ Femur length	4.877	<0.001
	SM length ~ Femur length	15.397	<0.001
	IFib length ~ Femur length	4.065	<0.001
	SA length ~ Femur length	4.322	<0.001
	AM (ventral head) length ~ Femur length	13.42	<0.001
	GRM length ~ Femur length	12.921	<0.001
	TiP mass ~ Tibiofibula length	3.1	0.004
	PER mass ~ Tibiofibula length	−4.24	<0.001
	TiaL mass ~ Tibiofibula length	−3.812	<0.001
	PL length ~ Tibiofibula length	7.982	<0.001
	TiP length ~ Tibiofibula length	5.472	<0.001
	PER length ~ Tibiofibula length	9.002	<0.001
	ECB length ~ Tibiofibula length	3.862	<0.001
	TiAB length ~ Tibiofibula length	3.163	0.0037
	TiAL length ~ Tibiofibula length	9.528	<0.001

*Note*: Any combinations not included in this table did not show any significant correlation. The full names of each muscle can be found in Figures 1 and 2 and Table 1.

**FIGURE 10:**
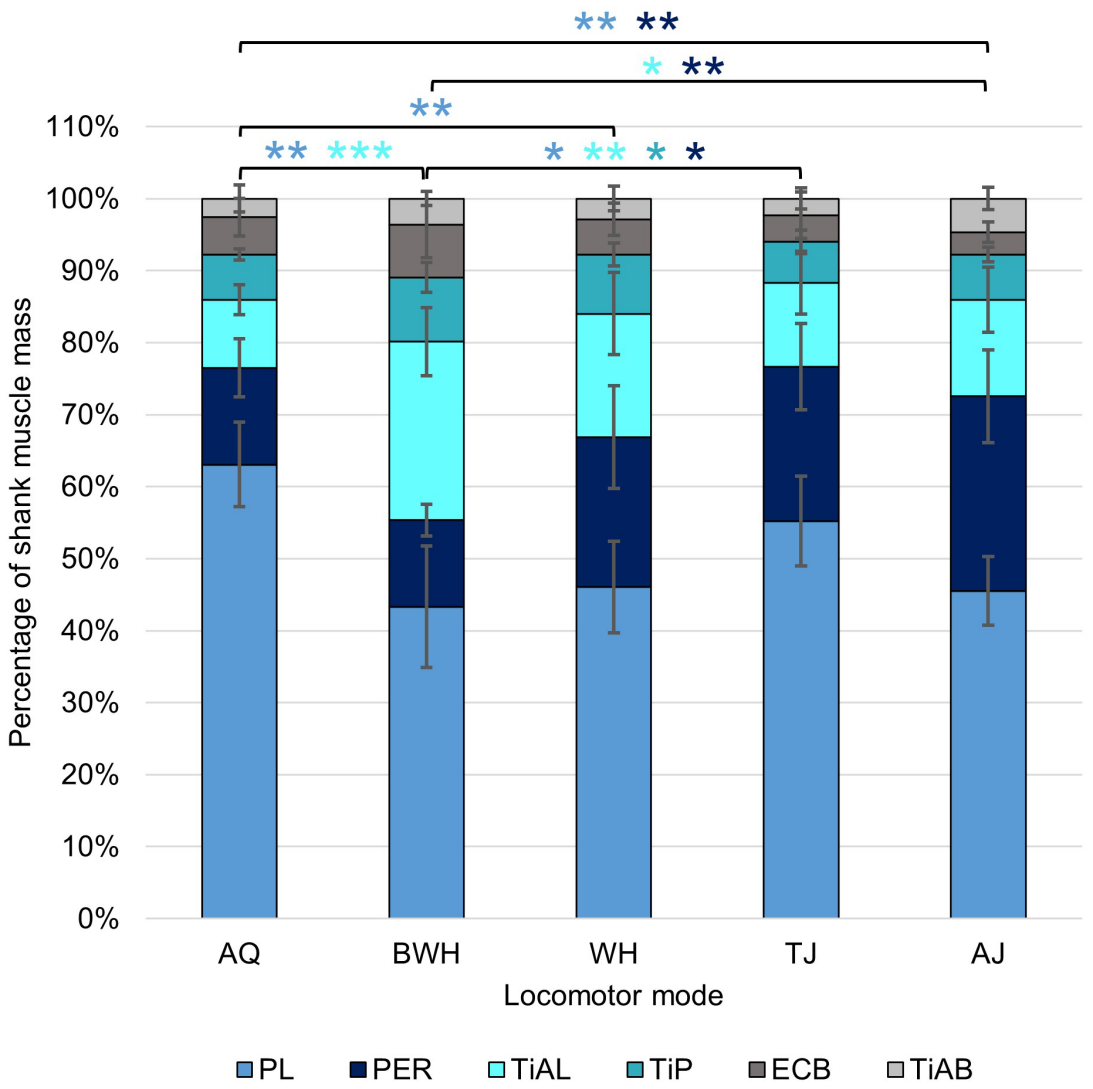
Average mass across locomotor modes for muscles in the shank prior to log transformation. The error bars represent standard deviation from the mean. Tukey test significance values refer to the log‐transformed variables and are represented by ***p < 0.001, **p < 0.01 and *p < 0.05. There are no significant differences between locomotor modes for the ECB when controlled for phylogeny. ECB, extensor cruris brevis; PER, Peroneus; PL, Plantaris longus; TiAB, Tibialis anticus brevis; TiAL, tibialis anticus longus; TiP, tibialis posticus.

**FIGURE 11:**
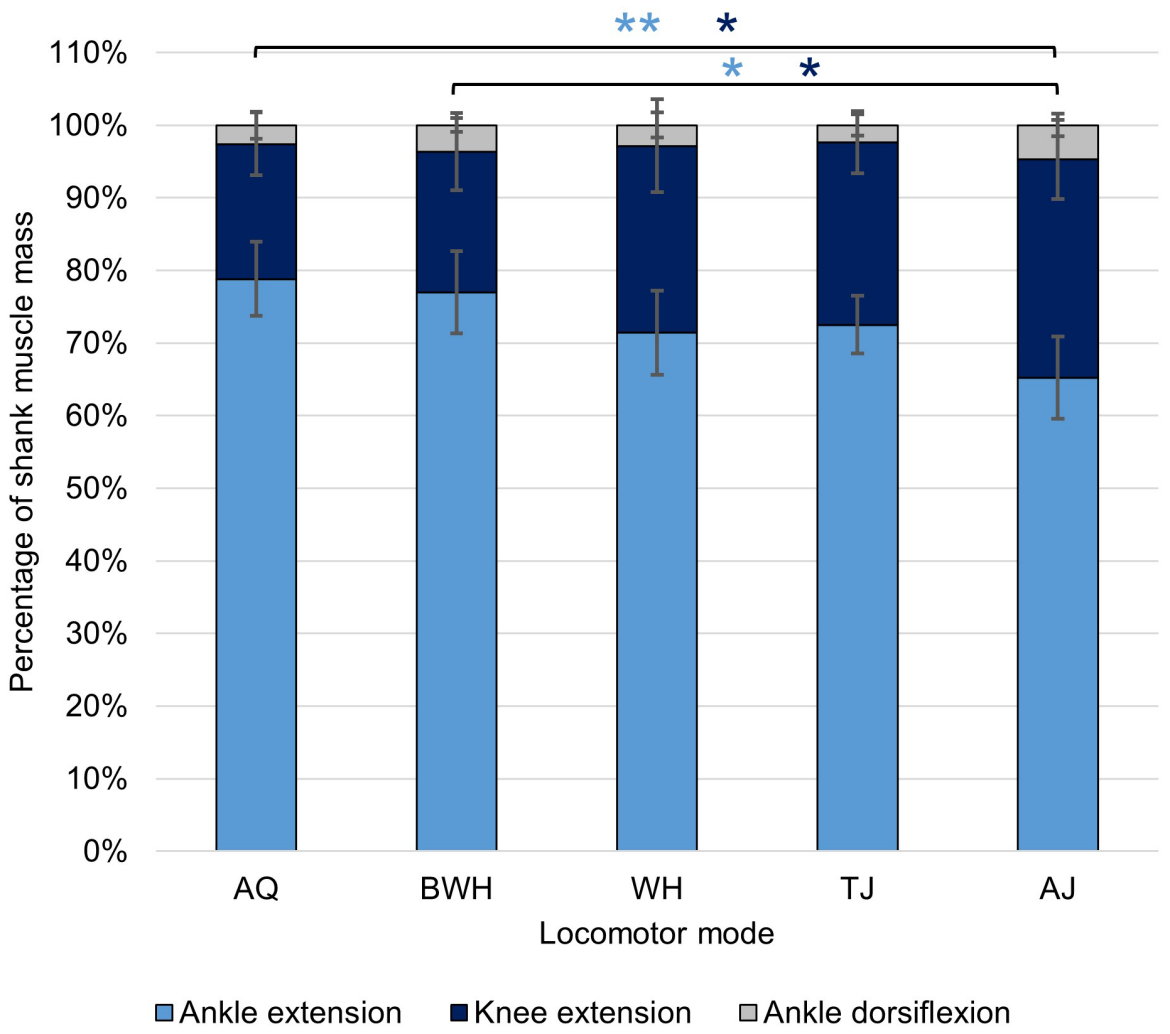
Average mass of each functional muscle group in the shank prior to log transformation. The error bars represent standard deviation from the mean. See Table 1 for the muscles within each functional group. Colours match the muscle in Figure 10 that contributes most to each functional group. Tukey test significance values refer to the log‐transformed variables and are represented by **p < 0.01 and *p < 0.05.

The shank shows the most correlations between tibiofibula length and the mass and length of each of its associated muscles (Table 3). The shank is also the segment with the most significant differences between locomotor modes in muscle lengths and masses. TJ, AJ and AQ consistently present significantly longer shank muscles than BWH (Table S3). AJ has the smallest total amount of variation in shank muscle anatomy, while WH has the largest (Figures 8 and 10). The first four pPC axes explain 95.81% of the total variance (Table S4). Shank muscle composition is primarily determined by the relative mass of the extensor cruris brevis (ECB) and peroneus in pPC1 and the tibialis anticus brevis and plantaris longus in pPC2 (Figure 8). For pPC1, BWH and WH values are generally higher than AJ, TJ and AQ as they have a considerably larger ECB. pPC2 values are smaller in AQ and TJ compared with BWH and AJ (Table 2), largely due to their larger plantaris longus muscle (Figure 8). There are no significant differences between phylogenetic groups along either axis. For the shank functional muscle groups, none demonstrate a phylogenetic signal (Table S2). Ankle extensors (ANOVA: *F*_(4,25)_ = 5.00, *p* = 0.004) and knee extensors (ANOVA: *F*_(4,25)_ = 4.34, *p* = 0.008) show significant differences between locomotor modes. AJ have significantly smaller ankle extensors than both AQ and BWH (Figure 11). AJ instead has significantly larger knee extensors than both AQ and BWH.

**FIGURE 12:**
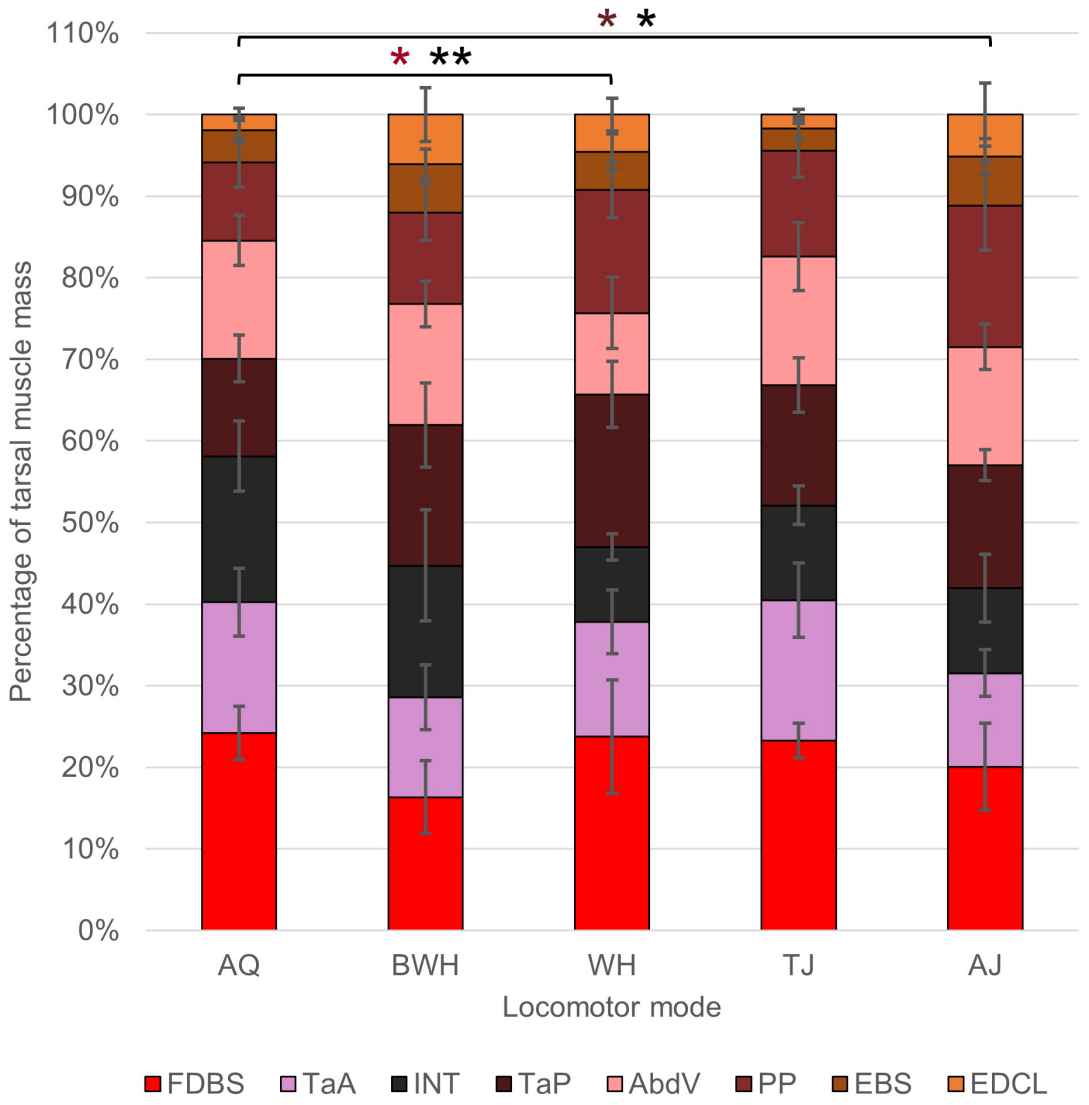
Average mass across locomotor modes for muscles in the proximal foot prior to log transformation. The error bars represent standard deviation from the mean. Pairwise phylANOVA (INT) and Tukey test significance values refer to the log‐transformed variables and are represented by **p < 0.01 and *p < 0.05. AbdV, adductor brevis dorsalis V; EBS, extensor brevis superhallucis; EDCL, extensor digitorum communis longus; FDBS, flexor digitorum brevis superficialis; INT, intertarsalis; PP, plantaris profundus; TaA, tarsalis anticus; TaP, tarsalis posticus.

Regarding the proximal foot segment, the extensor brevis superhallucis (EBS) is the only muscle that does not have a length positively correlated with calcaneus length (Table 3), supporting Hypothesis 1. The mass of the flexor digitorum brevis superficialis is also positively correlated with calcaneus length, while the intertarsalis shows a significant negative correlation (Table 3). As with the other hindlimb segments, BWH have significantly shorter proximal foot muscles than the other locomotor modes (Table S3). Proximal foot muscle composition is primarily determined by the relative mass of the adductor brevis dorsalis V and extensor digitorum communis longus (EDCL) in pPC1, and the EBS and EDCL in pPC2 (Figure 8). The first four pPCs explain 86.18% of the variance (Table S4). WH has the most variation in proximal foot muscle anatomy, while AJ has the least (Figure 12). None of the locomotor modes nor phylogenetic groups are significantly different from each other when *p‐*values are corrected for multiple testing, though TJ and AQ generally have larger pPC1 values than AJ and WH (Table 2).

### 3.4 Muscle head number

All information on muscle head number is available in the Supplementary Dataset. None of the best fit models included voxel size as an explanatory factor, meaning that scan resolution did not significantly impact muscle head number (Table S7). The pelvis, thigh and shank usually contain 5, 17 and 6 post‐vertebral muscles respectively, which aligns with previous findings (Collings & Richards, 2019; Přikryl et al., 2009). Regarding pelvic muscle head number, phylogenetic signal appears high, but not significantly different from zero (Table S2). Essentially, the only differences in the degree of muscle separation in the pelvis in the present study are for the coccygeosacralis (absent in *Alytes obstetricans*, *Barbourula busuangensis*, *Breviceps poweri*, *Sechellophryne gardineri and Xenopus laevis*), the iliacus externus (four layers in *X. laevis*, two heads in *A. obstetricans*) and the pyriformis (missing in *X. laevis*; Porro & Richards, 2017). There are no significant differences between locomotor modes, suggesting a lack of support for Hypothesis 4.1.

**FIGURE 13:**
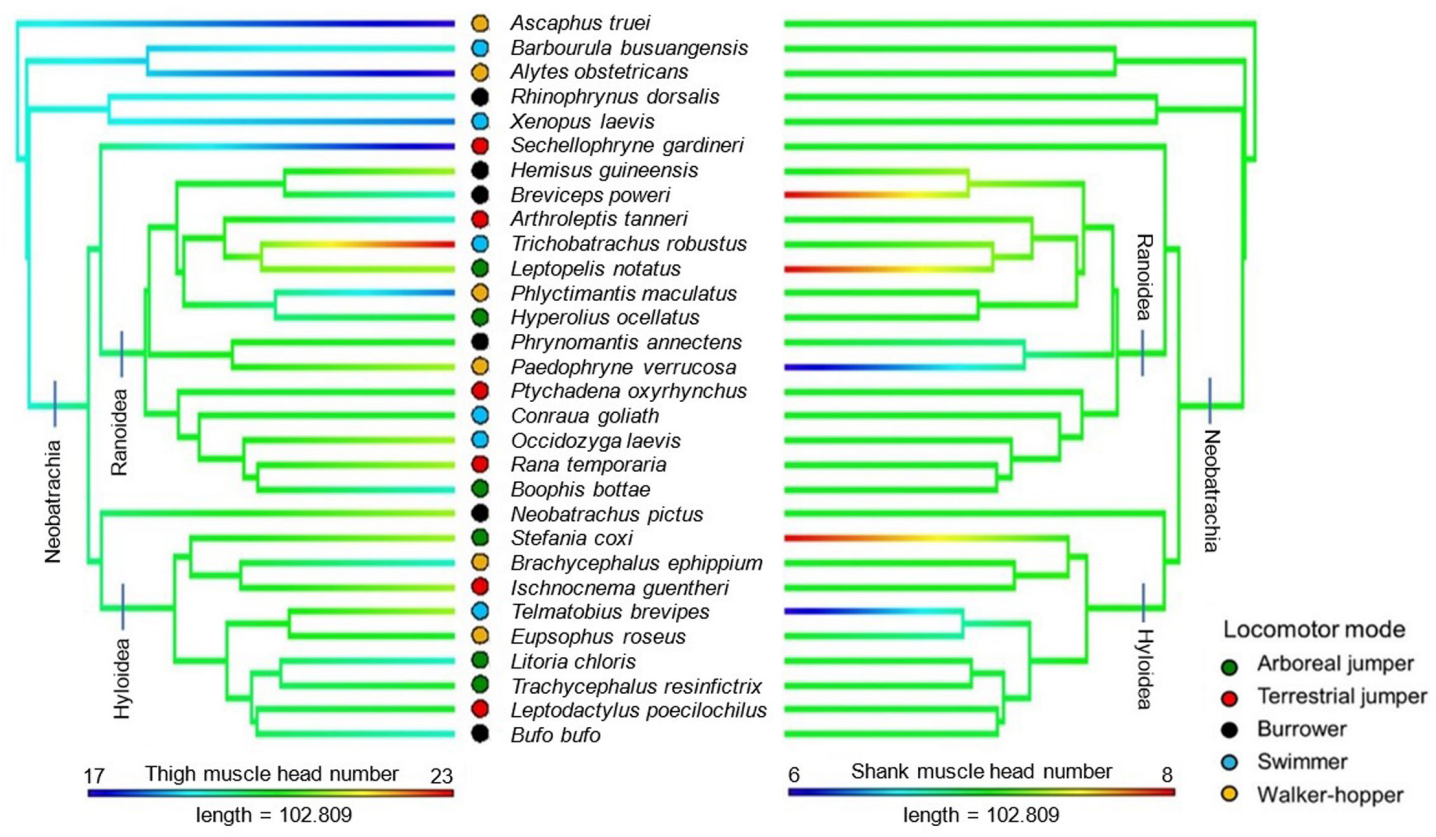
Phylogeny of study taxa derived from Jetz and Pyron (2018) where branch colours represent muscle head number for the thigh (left) and shank (right).

In the thigh, the separation of muscles into distinct parts occurred for all species in the adductor magnus (dorsal and ventral heads). The next most common cases of muscle separation occurred in the adductor longus (distinct from the pectineus in 20 species), semitendinosus (dorsal and ventral heads in 19 species) and cruralis (deep and superficial layers in 14 species). Additionally, the tensor fascia latae is missing in *B. poweri* and *Hyperolius ocellatus*, while there are two distinct heads in *Occidozyga laevis*. In *Ptychadena oxyrhynchus* and *Stefania coxi*, the semimembranosus appeared to have two very distinct heads rather than the oblique tendinous inscription described in previous studies (Collings & Richards, 2019), but these cases were not counted for analysis since the tendons could not be visualised to confirm this. The gracilis minor is not distinguishable from the gracilis major in *Sechellophryne gardineri*. Thigh muscle head number is more variable compared with the pelvis and shank, ranging from 17 to 23 muscle heads, and is often lower in Archaeobatrachia (Figure 13). There are no significant differences in thigh muscle head number between locomotor modes.

Muscle head number is the most uniform in the shank (Figure 13). The tibialis anticus longus is separated into two distinct heads for all species excluding *Paedophryne verrucosa* and *Telmatobius brevipes*. In line with previous studies (e.g. Collings & Richards, 2019), this muscle varied greatly in the point at which the muscle belly splits, and how much the heads differ in size. For example, *Litoria chloris* has the greatest size difference (0.28:1), while *S. scalae* has the smallest (0.99:1). The extensor cruris brevis and the plantaris longus have only one case each where the muscles are separated into two distinct heads (*Leptopelis notatus* and *S. scalae*, respectively). The shank muscle head number is the only variable in this analysis to show significant differences between locomotor modes (Table 4) showing support for Hypothesis 4.1.

**TABLE 4:** PGLS coefficients for shank muscle head number.

Coefficients	Estimate	Standard error	*t*‐value	*p‐*value
Intercept	7.333	0.16	45.873	**<0.001**
AQ	−0.5	0.226	−2.212	**0.036**
BWH	−0.167	0.226	−0.737	0.468
TJ	−0.333	0.226	−1.474	0.153
WH	−0.5	0.226	−2.212	**0.036**

*Note*: The *p*‐values in bold highlight significance values below the 0.05 threshold.

## 4 DISCUSSION

The present study aimed to relate the muscular anatomy of the pelvis and hindlimb in frogs to their locomotor mode and their evolutionary history to enhance our understanding of the relationship between form and function in vertebrates. This detailed study of anatomical structures aimed to address four hypotheses, three of which investigate variation in muscle sizes, while the other one examines differences in muscle separation. DiceCT has shown that species exhibiting different locomotor modes differ significantly in the size of many small hip and shank muscles, providing novel evidence of their functional significance. The current paper also marks the first quantitative analysis of how the degree of muscle separation can differ between frogs. Our main findings are as follows: (1) In general, bone length can sometimes reliably predict muscle length, but not muscle mass; (2) in general, pelvic crest lengths are not reliable predictors of pelvic muscle sizes, but are instead shaped more by evolutionary history; (3) the distribution of muscle mass among hindlimb segments varies depending on locomotor mode; (4) phylogenetic history appears to be the key driver for muscle separation in the pelvis and thigh (rather than locomotor mode); and (5) the number of separate shank muscle heads is influenced more strongly by locomotor mode (rather than phylogenetic history). Additionally, we have provided the 3D anatomical reconstructions of the pelvis and hindlimbs required for future biomechanical simulations to determine what consequences the observed variation in muscle size and intramuscular separation could have for the functional workspace of the limb.

### 4.1 Bone size is not always an appropriate proxy for muscle size

Palaeontological studies infer behaviour from fossils by using the shape and size of bones to estimate the size and attachment sites of muscles (Bates et al., 2021). Our study examines the relationship between muscle mass and length and the length of their associated long bones (Hypothesis 1). In addition, we investigate whether the length of the dorsal crests on the ilia and urostyle are associated with larger pelvic muscles (Hypothesis 2). To summarise, there is mixed support for both Hypotheses 1 and 2, suggesting that the shape of several bones is not appropriate proxies for estimating muscle mass and therefore functional properties. For example, there are no significant relationships between the mass of any pelvic muscles and the length of their associated bones, contrasting with Hypothesis 1. Regarding the hindlimb, while the shank shows a significant, positive relationship between total muscle mass and tibiofibula length, the thigh and proximal foot do not. However, there are many correlations among the length of the hindlimb bones and the mass and length of individual hindlimb muscles (Table 3), showing support for Hypothesis 1. This includes all the small shank muscles, the small hip muscles and the iliofibularis, supporting a previous suggestion that small hindlimb muscles can play a crucial role in shaping bone design (Vera et al., 2022). Ultimately, however, there is likely another functional explanation besides muscle size for why there are significant differences in bone lengths between locomotor modes (Leavey et al., 2023). For example, slight alterations in hindlimb proportions could cause subtle differences in segment rotations, which might have large effects due to known impacts of bone posture on muscle moment arm and muscle function (Collings et al., 2022). Direct mechanical evidence for how variations in frog hindlimb proportions influence locomotor mechanics is currently lacking and should be explored in future studies.

Regarding Hypothesis 2, the only muscle which had a significant positive relationship between dorsal crest length and muscle mass is the coccygeosacralis, which is responsible for bending or stiffening the trunk during locomotion (Přikryl et al., 2009). Additionally, there are no correlations between crest lengths and the length of any of the pelvic muscles despite positive correlations between iliacus externus length versus iliac shaft length and coccygeoiliacus length versus urostyle length (Table 3). Therefore, the length of the pelvic crests is unlikely to strongly impact the size of the pelvic muscles that attach to these areas. This lack of support for Hypothesis 2 could be because crest length alone is not sufficient to infer muscle attachment area, and resulting muscle size and/or locomotor function for this sample size. The height or area of the crests could be a more meaningful proxy for muscle size and locomotor function, for example. Additionally, some species (e.g. *Ascaphus*, *Barbourula*, *Paedophryne and Sechellophryne*) have lateral urostylic ridges which are not analysed here that might instead provide scaffolding for larger pelvic muscles. Alternatively, there may be other drivers for variation in the shape of osteological crests besides variation muscle structure. Our evolutionary models suggest that crest length for both the ilia and urostyle is shaped significantly by phylogenetic history, where more recently derived taxa tend to have larger crests (Table S2; Emerson, 1979; Leavey et al., 2023; Ponssa et al., 2018; Reilly & Jorgensen, 2011). However, our study found no significant differences between locomotor modes in terms of crest lengths, nor the size of the muscle‐crest attachment sites, which have previously been shown to differ most strongly between jumpers to walkers (Ponssa et al., 2018). The role of iliac and urostylic crests in locomotor function needs to be analysed in more detail using direct functional studies (see Section 4.11).

### 4.2 There is broad morphological variation in the shape and size of pelvic muscles

In line with previous studies (Collings & Richards, 2019; Fabrezi et al., 2014; Přikryl et al., 2009), we find pelvic myology to be highly variable, displaying a wide range of origins, insertions and sizes. Ultimately, there are no significant pairwise differences in pelvic muscle masses and lengths between locomotor modes. This lack of significant differences between locomotor modes may be because the pelvic muscles have equally relevant roles in all locomotor behaviours. For instance, despite the shape of the sacral bone driving the most variation in post‐vertebral skeletal anatomy (Buttimer et al., 2020; Emerson, 1982; Leavey et al., 2023; Petrović et al., 2017; Reilly & Jorgensen, 2011), there is little variation in the muscles with the largest sacral attachment sites—the longissimus dorsi and coccygeosacralis. Both these muscles are important for lateral bending during walking (Collings & Richards, 2019), fore‐aft gliding during swimming and extension of the sacrum during the initial jump phase (Ponssa et al., 2018), as they function by dorsally rotating the urostyle and bending or stiffening the trunk, respectively (Přikryl et al., 2009). In general, the longissimus dorsi tends to be longer in non‐jumpers, but has the largest mass in TJ, while the coccygeosacralis is, on average, longest in walkers (Figure S3), but most massive in swimmers (Figure 4). This could reflect how longer muscles tend to permit a wider range of motion (i.e. lateral bending during walking), while muscles with a higher volume are capable of generating more force (i.e. for jumping and swimming) (Lieber & Bodine‐Fowler, 1993). The coccygeoiliacus, which is responsible for rotating the urostyle ventrally and gliding the ilia anteriorly along the sacral diapophyses, is consistently the largest of the post‐vertebral pelvic muscles and yet it shows no obvious groupings of locomotor functions (Figure 4), again implying that this muscle might support multiple functions. As the lateral and long‐axis rotator of the urostyle, the pyriformis is said to be involved in multiple functions (Přikryl et al., 2009), though it is generally much larger in walkers and burrowers.

The iliacus externus is, on average, longer in jumpers and swimmers (Figure S3) and considerably larger in Archaeobatrachia than more phylogenetically derived taxa (Table 2; Figure 5), supporting the phylogenetic analysis by Fabrezi et al. (2014). This muscle varies widely in its functional capabilities depending upon its length and hindlimb posture (Přikryl et al., 2009), but is generally essential as a femur protractor and hip flexor for the swing phase of walking and climbing (Collings & Richards, 2019), recovery phase of swimming and crouched position in jumping (Nauwelaerts et al., 2007). The lack of significance in the size of the iliacus externus between locomotor modes could also be explained by an observation made by Collings and Richards (2019)—that the functional implications of the iliacus externus could be related more to its shape than its size. As longer muscles allow a greater range of motion (Lieber & Bodine‐Fowler, 1993), while shorter muscles with a larger volume generally result in higher cross‐sectional area and force output, there could be a trade‐off in the shape of the iliacus externus relating to locomotor function. In *Phlyctimantis maculatus*, for example, the iliacus externus is long and rather cylindrical, affording it the range of motion required to bring leg upwards and forwards while running (Collings & Richards, 2019).

### 4.3 Hindlimb segment muscle mass reflects different functional demands among locomotor modes

The present study investigated whether the total muscle mass invested into each segment of the hindlimb differs across locomotor modes in frogs (Hypothesis 3). If muscle mass simply increases with the relative length of the limb segment, then it would be expected that swimmers would invest the most muscle mass in the thigh, while jumpers would invest the most in the shank and proximal foot (Table 2). Similarly, walkers would have the smallest relative thigh muscle mass, and burrowers have the smallest relative shank and proximal foot mass. However, we found that walkers have the highest relative thigh muscle mass, followed by burrowers, swimmers and jumpers (Figure 6). Since the present study examines relative proportions, this does not necessarily mean that WH have stronger thighs than other locomotor modes; they may just not invest as much muscle into their other segments. In alignment with our expectations, jumpers and swimmers invest most strongly into shank musculature (Figure 6), driven primarily by the large size of the plantaris longus due to the strong requirements for toe and ankle entension (Figures 8 and 10; Přikryl et al., 2009; Vera et al., 2022). Despite their short tarsals, burrowers invested more muscle mass into this segment, presumably to increase the forces required for scooping dense substrates (Vidal‐García et al., 2014). However, it is also worth noting that the only significant difference in total hindlimb muscle distribution is that AJ have a larger relative shank muscle mass than WH. Since total hindlimb muscle distribution is statistically similar across all locomotor modes, this could be an example of one‐to‐many mapping of form of function in frogs (Moen, 2019; Wainwright, 2005).

### 4.4 Variation in the mass of the largest muscles is not sufficient to predict locomotor function

The cruralis is the most well‐studied thigh muscle and has been described as the functional mediator between jumping and swimming (Astley, 2016; Danos & Azizi, 2015; Garcia‐Pelagio et al., 2023; Gillis & Biewener, 2000; Marsh, 2022; Nauwelaerts et al., 2007; Peplowski & Marsh, 1997). As the largest and most pinnate muscle in the thigh (Calow & Alexander, 1973; Nauwelaerts et al., 2007, Přikryl et al., 2009, Figure 7), the cruralis generates considerably large forces important for jumping and swimming (Astley, 2016; Gillis & Biewener, 2000). As expected, the cruralis is the strongest driver of myological variation, particularly for TJ and burrowers (Figure 8). The knee extension group (cruralis, gluteus magnus, tensor fascia latae) is also larger in TJ and swimmers compared with walkers (Figure 9). It was expected that this functional group would also be small in burrowers, since the only large difference between jumping and burrowing is supposedly the asynchronous movement of the hindlimbs and the lack of femur extension during burrowing (Emerson, 1976), but this is not the case. Additionally, given that the primary selection pressure acting on jumping is predation (Nauwelaerts et al., 2007), while climbing/walking is primarily a method for traversing the canopy, it is surprising to find that AJ have significantly smaller knee extensors (Figure 7). An enlarged cruralis and gluteus magnus might somehow impede the function of other thigh muscles which are important for climbing/walking, but functional analyses would be needed to test this.

The adductor magnus, gracilis major and semimembranosus are the next largest thigh muscles (Figure 7; Gillis & Biewener, 2000; Nauwelaerts et al., 2007) and are responsible for femur protraction, adduction and retraction (Přikryl et al., 2009). Femur protraction is important for obtaining the crouched position prior to jumping and reducing the recovery phase during swimming (Astley, 2016; Nauwelaerts et al., 2007; Přikryl et al., 2009), while efficient femur retraction is vital for power amplification during jumping, which is particularly important for small frogs (Astley & Roberts, 2014; Roberts & Marsh, 2003). Adduction has been linked to jumping performance, while abduction is important for swimming (Nauwelaerts et al., 2007). While there are some small significant differences between some locomotor modes for the adductor magnus (smaller in burrowers than TJ and WH) and semimembranosus (smaller in burrowers than AJ; Figure 7), there are no significant differences between locomotor modes for any of the functional groups these large muscles occupy (Figure 9). The lack of significant differences in these evidently important thigh muscles suggests that either all locomotor modes require all of these functions to a similar extent, or anatomical/physiological muscle properties besides just relative mass and overall length need to be considered. For example, the femur retractors in many jumping mammals have a more proximal insertion onto the tibia (Emerson, 1985), and the fibre lengths and degree of pennation for each muscle differ across frog species (Kargo & Rome, 2002; Rabey et al., 2015; Astley, 2016; Leavey et al., *in review*).

The plantaris longus is the most well‐studied shank muscle, and the most frequently used hindlimb muscle for quantifying how contractile properties vary with locomotor performance (Astley, 2016; Azizi & Roberts, 2010, 2014; Clemente & Richards, 2013; Garcia‐Pelagio et al., 2023; James et al., 2005; James & Wilson, 2008; Marsh, 2022; Mendoza & Azizi, 2021; Richards & Biewener, 2007; Richards & Clemente, 2013; Roberts et al., 2011; Roberts & Marsh, 2003; Sawicki et al., 2015; Wilson et al., 2004). Its large mass, pinnate fibre architecture and long tendon have all been correlated with variation in jump performance (Azizi & Roberts, 2014; James et al., 2005; Marsh, 2022; Roberts et al., 2011; Roberts & Marsh, 2003; Sawicki et al., 2015). Additionally, it is known to have long electromyographic activity bursts important for the propulsive phase of swimming and for balancing hydrodynamic forces while the foot rotates (Astley, 2016; Gillis & Biewener, 2000; Richards & Biewener, 2007; Richards & Clemente, 2013). The plantaris has even been shown to vary across two populations of the same invasive species, where frogs at the edge of the locality invest in larger, more pinnate ankle extensors as an adaptation for range expansion (Padilla et al., 2019). In the present study, swimmers have considerably larger ankle extensor muscles than AJ, burrowers and walkers (Figure 10), driven primarily by the size of the plantaris longus (Figures 8 and 11). Swimmers also showed the smallest amount of variation in shank anatomy (Figures 8 and 10), implying that there are strong selective pressures to conserve shank muscle composition. This aligns with previous work by Richards (2010), who found that swimmers rely more on rotational thrust powered by the ankle than translational thrust powered by the thigh musculature. Despite the importance of the plantaris longus in determining jump performance in tree frogs (Mendoza & Azizi, 2021; Roberts et al., 2011), AJ unexpectedly have significantly smaller ankle extensors than burrowers and swimmers (Figure 11). However, this does not necessarily mean that arboreal taxa are bad at jumping. The plantaris longus, peroneus, tibialis posticus and tibialis anticus longus are all longer on average for AJ compared with the other locomotor modes, especially burrowers (Table S3; Figure S5). Additionally, unlike TJ, AJ invest muscle mass into a significantly larger knee extension group in the shank rather than the thigh, driven primarily by the peroneus (Figure 11). These differences in the distribution of muscle mass throughout the hindlimb between jumpers may be because arboreal taxa need to compensate for a displaced centre of gravity (De Oliveira‐Lagôa et al., 2019), have the ability to climb as well as jump (Simons, 2008) and account for differences in substrate compliance (Reynaga et al., 2019).

### 4.5 Small muscles may have an underappreciated role in locomotor function

There is very little information in the literature about the significance of the smaller muscles in the thigh and shank (Vera et al., 2022), particularly those near the hip due to the difficultly associated with extracting them intact using traditional dissection methods. Our research presents novel evidence that small hindlimb muscles can differ significantly in size between locomotor modes. This likely represents the different strategies employed by each locomotor mode in how they modulate the function of the large muscles. For example, the muscle which stabilises the femur, the pectineus and the muscles important for knee extension, such as the cruralis, are significantly larger in TJ compared with both walkers and AJ (Figure 7). A larger pectineus may be important in terrestrial jumping to influence the position of the femur with relatively little force, and hence alter the moment arm, and therefore function, of the thigh knee extensors with greater efficiency (Figure 9). Meanwhile, the muscle responsible for ankle dorsiflexion, the tibialis anticus brevis, is considerably larger in AJ compared with TJ and swimmers (Figure 11). These instances also provide another example of the impact of differences in habitat requirements on hindlimb myology.

The obturator internus, the muscle responsible for long‐axis rotation of the femur, is significantly smaller in burrowers than the other locomotor modes, supporting Hypothesis 3 and suggesting that there is a less important function for this muscle in burrowing. This, and the significant differences across the principal components of the thigh (Figure 8), contrasts with Emerson's (1976) hypothesis that thigh modifications for jumping are suitable exaptations for burrowing. The structure of small shank muscles in burrowers is also unique. The extensor cruris brevis, which is part of the knee extension group, inserts more distally onto the tibiofibula in burrowers compared with jumpers (Figure S5). This is said to increase the amount of force generated at the distal end of the shank during knee extension and lateral rotation (Emerson, 1976). Similarly, the tibialis anticus longus always had two very distinct heads in burrowers (Supplementary Dataset), which has been suggested to increase the force of ankle extension without involving movement of the hip, unlike the other muscles in this functional group (Emerson, 1976). The importance of these two functions is supported by their considerably larger size in burrowers (Figure 10) and the tighter clustering of burrowers on the shank pPC plot (Figure 8) compared with the other locomotor modes. This important variation in shank composition cannot be observed when the tibialis anticus longus is grouped with the other ankle extensors (Figure 11). This highlights how one muscle alone should not be used to represent the functionality of a limb, as a functional group may not have a completely synergistic influence on locomotor function. Functional analyses will be needed to directly determine how much the variation in these small muscles can impact behaviour.

### 4.6 Burrowing style does not noticeably impact pelvic and hindlimb anatomy

Prior to this study, there was a noticeable a lack of research into the differences in post‐vertebral musculoskeletal anatomy between different types of burrowing frogs. Interestingly, forward burrowers (*Hemisus guineensis*, *Rhinophrynus dorsalis*) are not clustered separately from the backward (*Breviceps poweri*, *Neobatrachus pictus*), and non‐descript (*Bufo bufo*, *Phrynomantis annectans*) burrowers for both the pelvic (Figure 5) and hindlimb muscles (Figure 8). Since backwards burrowing is the basal condition (Nomura et al., 2009) and prevalent in ~95% of burrowing frogs (Emerson, 1976), these results suggest that changes in the forelimbs and pectoral girdle may be all that differentiates forward burrowers from their ancestral condition (Engelkes et al., 2020; Keeffe & Blackburn, 2020, 2022). Unfortunately, there is no information on the exact function of pelvic muscles for burrowing in the literature, making this an area worth studying in more detail.

### 4.7 The primary driver of variation in muscle head number depends on the hindlimb segment

Muscle head number may change in line with locomotor requirements (Hypothesis 4.1) or evolution (Hypothesis 4.2). The results of the present study indicate that these relationships differ between each part of the anuran anatomy. The pelvis and the thigh, which have the most variation in muscle head number, both have a higher degree of muscle separation in more derived taxa (Figure 13; Table 2), providing some support for Hypothesis 4.2. However, the moderate phylogenetic signals for both pelvic pPCA axes and the first thigh pPCA axis are not significant. Similarly, shank muscle head number and muscle size had no phylogenetic signal, but had a *p*‐value of one (Table S2). Without analysing this across a larger sample size (Münkemüller et al., 2012), we cannot conclusively say that evolutionary history impacts muscle size and separation in the pelvis and thigh, but not the shank. However, the shank does show significant differences between locomotor modes, providing evidence for Hypothesis 4.1 (Figure 13; Table 4). Specifically, walkers and swimmers are less likely to have an unseparated tibialis anticus longus, which functions as an ankle extensor. Although the number of muscle heads in the proximal foot is not evaluated, phylogenetic signal for muscle size is also zero and there is evidence of differences between locomotor modes in terms of muscle composition (Figure 8). Therefore, our results support Hypothesis 4.1, as well as the findings of a previous study, which suggests that the muscle architecture of more distal limbs segments is more labile across evolution and is more closely correlated to locomotor performance (Astley, 2016). The proximal‐distal sequence of increasing variation in muscle composition across segments found in the present study further supports this hypothesis (Figure 8).

### 4.8 Understanding the functional trade‐offs associated with muscle separation will facilitate our understanding of how anatomical complexity is related to functional complexity

Muscle separation is thought to contribute towards more precise motor control and to create a larger area of functionality within which the limb can perform as it allows for separate nerve innervation and an increase in the range of external moment arms (Collings & Richards, 2019; Gans & Bock, 1965). Therefore, we would expect that a locomotor generalist, such as *Phlyctimantis maculatus* (Ahn et al., 2004; Danos & Azizi, 2015), would have the most muscle separation to achieve such a wide range of functions, either due to a culmination of abilities through its ancestors, or a set of recent adaptations in response to new environmental conditions (McShea & Hordijk, 2013). However, we find that *P. maculatus* has an average shank muscle head number and the lowest thigh muscle head number compared with all of the species in the more phylogenetically derived groups, Hyloidea and Ranoidea (Figure 13). In this case, it appears that anatomical complexity is not a prerequisite for functional complexity.

Furthermore, we find several cases where muscles undergo subsequent fusion (e.g. the tibialis anticus longus in swimmer *Telmatobius brevipes* and walker‐hopper *Paedophryne verrucosa*; Figure 13), or where entire muscles are lost (e.g. the tensor fascia latae in burrower *Breviceps poweri* and arboreal jumper *Hyperolius ocellatus*, two distantly related species; Supplementary Dataset). As muscle is an energetically expensive tissue (Perry & Prufrock, 2018), the subsequent fusion or loss of muscle heads could implicate that there has been a reduction in anatomical complexity to allow for more efficient function (McShea & Hordijk, 2013). However, this logic contradicts our findings for muscle head number in *P. maculatus*. Ultimately, these results raise the question: What functional trade‐offs are associated with muscle separation? More precise definitions of locomotor capabilities, biomechanical tests (see Section 4.11) and targeted phylogenetic approaches, such as ancestral state reconstruction (Astley, 2016), could be used to answer this question in future studies.

### 4.9 Frogs demonstrate high anatomical complexity that could be linked to ‘many‐to‐many’ mapping of form to function

Ultimately, this study has shown that knowing the properties of a few large pelvic and hindlimb muscles is not sufficient to accurately predict locomotor function in frogs. Evidently, frogs can use many different, overlapping variations of bone and muscle anatomy to meet their performance requirements, as we have observed several examples of both many‐to‐one mapping (e.g. pelvic and proximal foot musculature) and one‐to‐many mapping (e.g. total hindlimb muscle distribution) of form to function (Wainwright, 2005). These kinds of complex, labile relationships have been suggested to alleviate functional trade‐offs and therefore allow for diversity in function, that is the ability to perform multiple locomotor modes, albeit sub‐optimally (Herrel et al., 2014; Kargo & Rome, 2002; Moen, 2019; Nauwelaerts et al., 2007; Soliz et al., 2017). Rather than anatomical and functional specialisation, having an intermediate phenotype which can adapt to multiple locomotor requirements may instead be favoured by natural selection (Gans, 1993; Nauwelaerts et al., 2007), especially in environments where conditions are in constant flux. To test whether the high anatomical complexity observed in this study demonstrates convergence onto an intermediate phenotype, more digital dissections will be needed to extract an accurate phylogenetic signal for muscle anatomy (Münkemüller et al., 2012), and Orstein‐Uhlenbeck models of evolution will be required to identify the adaptive optima for each locomotor mode (Moen et al., 2016). In addition, since multiple types of form‐function relationships are interacting simultaneously in frogs, there is likely to be a highly complex matrix of ‘many‐to‐many’ mapping dictating their evolutionary trajectory, that is multiple phenotypic traits are influencing multiple measures of performance (Bergmann & McElroy, 2014). A future study could import performance measures across all locomotor modes and the important morphological traits identified in this paper into an interspecific ‘F‐matrix’ to quantify the complexity of this system in frogs with more certainty (Bergmann & McElroy, 2014).

### 4.10 Limitations

Digitising museum specimens has been increasing in popularity over the last decade, resulting in large collections of 3D data in repositories such as MorphoSource and iDigBio. However, our research has highlighted the need for a change in the way specimen and CT data are currently recorded. Standard body size measurements at the time of capture (e.g. body weight) and information on the time between capture and fixation, fixation duration and ethanol storage duration were not available for most taxa. Consequently, the present study is limited to making interpretations from relative muscle mass measurements, as variation in the level of soft‐tissue shrinkage could not be reliably controlled for. Since total hindlimb muscle mass is strongly associated with locomotor mode (James et al., 2005; Moen, 2019; Nauwelaerts et al., 2007; Vera et al., 2022), dividing the variables examined in this study by total hindlimb muscle mass for normalisation, instead of total body mass, might be diluting the trends we have observed. Additionally, the mass of an individual muscle might be correlated to the mass of the synergists in the same segment or anatomical compartment. While the inclusion of rare, endangered and/or recently extinct museum specimens necessitates the use of previously preserved specimens (Leonard et al., 2021), future studies should aim to digitise the specimens that have been captured within a year of the original fixation to limit the amount of shrinkage caused from alcohol storage (Gignac et al., 2016). Additionally, all scans uploaded to digital repositories should be supplemented with metadata containing all preservation, staining and scanning parameters, as specimen measurements which enable body size corrections.

Iodine cannot stain tendons, so tendinous structures cannot be visualised or measured using diceCT, thus limiting the functional inferences that can be made from soft tissue. Tendinous attachments impact elastic energy storage, metabolic energy conservation, muscle power amplification and mechanical feedback mechanisms (Roberts & Azizi, 2011) and are therefore expected to show significant differences between locomotor modes. Abdala et al. (2018), for example, used electron microscopy to show that jumpers have collagen fibrils with a greater cross‐sectional area than WH, which could reflect the role of tendons to absorb forces during landing. Tendons also permit the locomotor system to function beyond the limits of isotonic muscle contraction, which is essential for the spring‐actuated jumping mechanism in small frogs (Mendoza & Azizi, 2021; Roberts et al., 2011; Sutton et al., 2019). Additionally, tendons can be very long, making the origin and insertion points of muscles hard to determine without supplementary traditional dissection (e.g. the iliofibularis; Figure S1).

### 4.11 Future directions

Due to the limitations of the present study, two large issues of anuran comparative anatomy remain unresolved. Firstly, what classifies as a locomotor ‘specialist’ and ‘generalist’ has not been consistently defined across the literature, which undermines the ability to define complexity in anatomy and function (McShea & Hordijk, 2013; Vassallo et al., 2021). Future studies will need to analyse the locomotor skills and limitations of both ‘generalist’ and ‘specialist’ species in their natural environments to be able to fully understand how new niches originate. For example, we are strongly in need of studies that record natural behaviour both in the field and in the laboratory to get measurements of performance which could be paired with detailed morphological measurements to get precise structure–function knowledge (Vassallo et al., 2021). Furthermore, even just the inclusion of a third categorical variable which allows species to be described as a ‘moderate specialist’ (e.g. species with two locomotor modes, such as the semi‐aquatic jumper *Rana temporaria*) would provide more insight into whether anatomical complexity is a prerequisite for functional complexity. Ideally, locomotor specialisation should be considered along a continuous spectrum once enough data are collected. We may then find, for example, that locomotor modes which require specialised anatomical features may influence the adaptive landscape more strongly than those with multiple anatomical solutions, in which case being in the centre of morphospace may not be representative of a ‘generalised’ anatomy. By investigating the concept of ‘generalist versus specialist’ further, future studies can see whether having a complex functional repertoire reduces or increases the need for a more complex anatomy across all areas of the anuran phylogeny.

Secondly, we often can only infer function from anatomical observations. A recurring theme in the present paper is recognising the need for direct functional tests to confirm the inferences made about locomotor mode from musculoskeletal anatomy. With six pelvic muscles and over 30 hindlimb muscles which all have specific functions contributing differently to locomotion (Kargo & Rome, 2002; Přikryl et al., 2009; this study), there are too many parameters to reliably untangle without comparative functional analyses. Frog anatomy has many instances of complex muscle pathways, making their line of action difficult to determine from records of only the origin and insertion sites (Collings & Richards, 2019; this study). The lack of mechanical independence between muscles also means that even small amounts of anatomical variation can result in large functional differences (Kargo & Rome, 2002). Moreover, frog hindlimbs move in three different planes simultaneously (Astley & Roberts, 2014; Collings et al., 2022; Porro et al., 2017; Richards et al., 2017), so predictions of muscle function cannot be made solely from static muscle topology. Function can also vary throughout the duration of the movement and largely depends on initial limb configuration, making the interactions between joint torques, muscle forces and joint angles across multiple structures highly complex (Collings et al., 2022; Kargo & Rome, 2002).

The current paper outlines several specific questions which these musculoskeletal dynamics models could address. For example, future studies of extinct taxa would benefit from knowing how variation in hindlimb proportions impacts muscle dynamics, so that we can better understand the relationship between bone size, muscle size and function. Additionally, there is currently very little information on how muscle activation varies in muscles besides the largest ones in the thigh and shank (Reynaga et al., 2019), especially for locomotor modes besides jumping and swimming. It would be particularly interesting to test how the function of previously untested muscles (e.g. the iliacus externus, small hip muscles and tibialis anticus longus) changes in response to different locomotor functions within the same species/individual or across differences in substrate compliance (i.e. arboreal versus terrestrial habitats; Astley et al., 2015; Reynaga et al., 2019). Computational models may be the way forward, since many muscles are too small for more invasive techniques such as electromyography, and long‐axis rotation and femur stabilisation can be difficult actions to quantify. As a generally understudied locomotor mode (Emerson, 1976; Keeffe & Blackburn, 2020, 2022), how burrower morphology differs from walkers, and how muscle activation differs in forward‐ and backward‐burrowers are lines of enquiry which would also benefit from functional analyses. Finally, muscle anatomy may be shaped by other non‐locomotor activities, such as feeding, grooming and reproduction, that should be investigated in future work.

Another area of particular interest is how the varying degrees of muscular separation found across anurans might impact locomotion, as it provides a testable example of how anatomical complexity may influence functional complexity. Muscle separation likely functions as a way of increasing the range of possible hindlimb motions, and thus functional versatility (Collings & Richards, 2019), but this remains untested in frogs. If muscle separation increases the ability to perform multiple tasks, the number of muscle heads relative to other species could be a potential indicator of locomotor specialisation. To discover whether the variation in muscle anatomy we observed across species represents a division of functional roles, dynamics models should be used to selectively add and remove muscle heads and/or tendinous insertions, then measure the resultant changes in muscle moment arms and joint torques. The priority muscles to test would be the cruralis, adductor magnus and semitendinosus thigh muscles, as well as the tibialis anticus longus and extensor cruris brevis shank muscles, as they show the most variation in muscle separation (Supplementary Dataset). The gracilis major and semimembranosus should also be tested, as they often display intramuscular separation (Přikryl et al., 2009). It is also relatively unknown how muscle separation impacts fibre architecture and therefore the trade‐off between muscle force and contractile speed (Collings et al., 2022). Ultimately, musculoskeletal models would provide more direct evidence of how differences in hindlimb muscle architecture affects hindlimb multi‐functionality and therefore versatility in locomotor function. The present study has provided the 3D anatomical reconstructions required for these future studies, as well as for use as educational resources.

### 4.12 Conclusion

The present study contributes to the building body of evidence that there is no unique combination of musculoskeletal characteristics for each locomotor mode (Fabrezi et al., 2014; Marsh, 2022; Přikryl et al., 2009; Vera et al., 2022). Ultimately, musculoskeletal anatomy varies in response to locomotor requirements, habitat type and phylogenetic history. There are several cases where there is an unexpected lack of significant differences between groups, contrasting the findings of previous anatomical records and functional experiments. These results indicate that most myological features serve multiple functions, reflecting the complex mechanics of anuran hindlimbs. Furthermore, the size and topology of muscles within each segment of the hindlimb are likely shaped by different selection pressures—the shank appears to be influenced more strongly by locomotor mode compared with the pelvis and thigh. These labile relationships between anatomy and function provide the means for species to be able to perform multiple locomotor modes, albeit sub‐optimally. In an everchanging world, natural selection may favour the resultant intermediate phenotype for its ability to adapt to different locomotor requirements and environmental conditions (Nauwelaerts et al., 2007). Furthermore, this work has highlighted a series of interesting new hypotheses to test with biomechanical simulations using the 3D musculoskeletal reconstructions we have provided, particularly for the muscles which discriminate between arboreal and terrestrial habitats for jumping, and between burrowers and non‐burrowers.

## AUTHOR CONTRIBUTIONS

All authors contributed towards the project design. A.L. and L.B.P digitally dissected the specimens and A.L. performed the data collection and analysis. A.L. drafted the manuscript, and all authors provided constructive feedback.

## FUNDING INFORMATION

This project was supported by the London Interdisciplinary Doctoral programme, which is funded by the Biotechnology and Biological Sciences Research Council [grant number BB/M009513/1]. Costs associated with staining and scanning several museum specimens were covered by the oVert project which is funded by the National Science Foundation (grant number 1701714).

## CONFLICT OF INTEREST STATEMENT

The authors declare no conflict of interest.

## Supporting information


Data S1.

Data S2.

## Data Availability

All of the data associated with this manuscript (FigShare DOI: https://doi.org/10.6084/m9.figshare.26357395), and all the anatomical reconstructions (https://sketchfab.com/aleavey/collections) will be made freely available upon publication of this manuscript.
