# Supplementary material for: Comparative muscle anatomy of the anuran pelvis and hindlimb in relation to locomotor mode
Source: J Anat. 2024 Aug 9;245(5):751–74. doi: 10.1111/joa.14122 (PMC11470798; doi:10.1111/joa.14122)
Supplement: Supplementary file 1 — Data S1. [file JOA-245-751-s001.docx]

##### SUPPLEMENTARY INFORMATION

##### Troubleshooting diceCT

The hindlimb of one specimen of *Rana temporaria* was dissected using traditional methods. This exercise was important considering that tendinous structures cannot be viewed using the same contrast-enhancing agents used to visualise muscles. The frog was retrieved by L.B.P., already expired with no signs of damage or disease. Dunlap (1960) and Přikryl *et al.* (2009) were used to identify each muscle. Muscles were photographed, removed, measured using digital callipers (both muscle belly length and the length of the muscle-tendon unit) and weighed. Any observations that would be useful to consider while carrying out the work in the present study were noted, such as those presented in Figure S1.


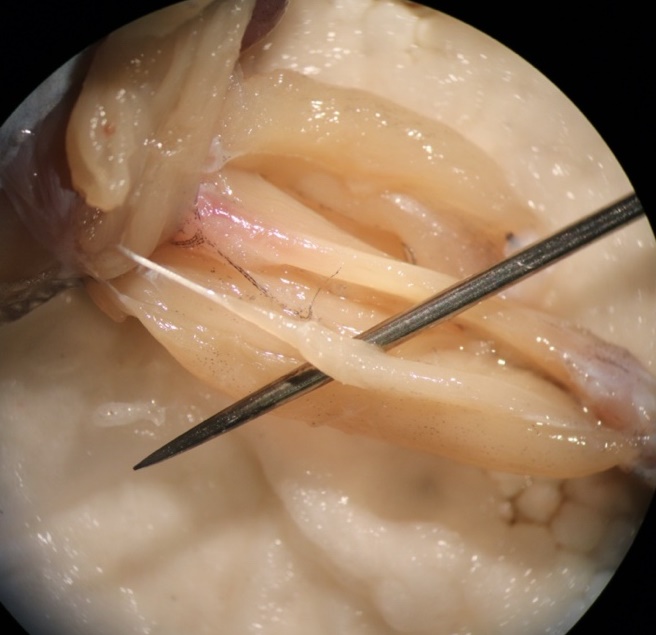

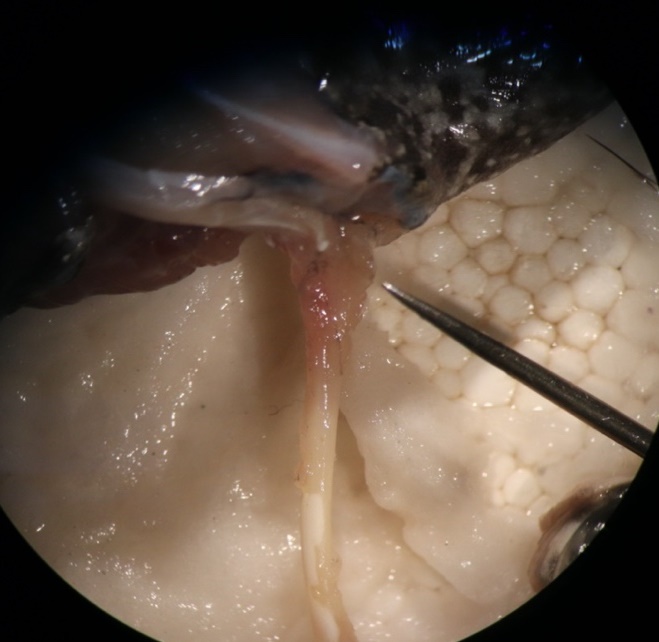


A)

B)

Figure S1 - Microscope images from a traditional dissection of *Rana temporaria*. A) The long tendinous attachment of the iliofibularis (red arrow) to its origin on the ilium can result in the underestimation of muscle-tendon unit length from diceCT alone. B) Several small hip muscles were not possible to dissect in-tact, such as the gemellus (green) and obturator externus (blue), highlighting the benefits of diceCT over traditional dissection.

We stained and scanned ten additional species, two of which were used to complete our dataset in terms of locomotor mode and phylogeny coverage. After the scan was complete, the specimens would be de-stained so that they could be returned to museum collections. Although the specimens appear almost identical to how they did before staining, µCT scans show that tissue can remain radio-opaque for many years after the experiment (Figure S2).


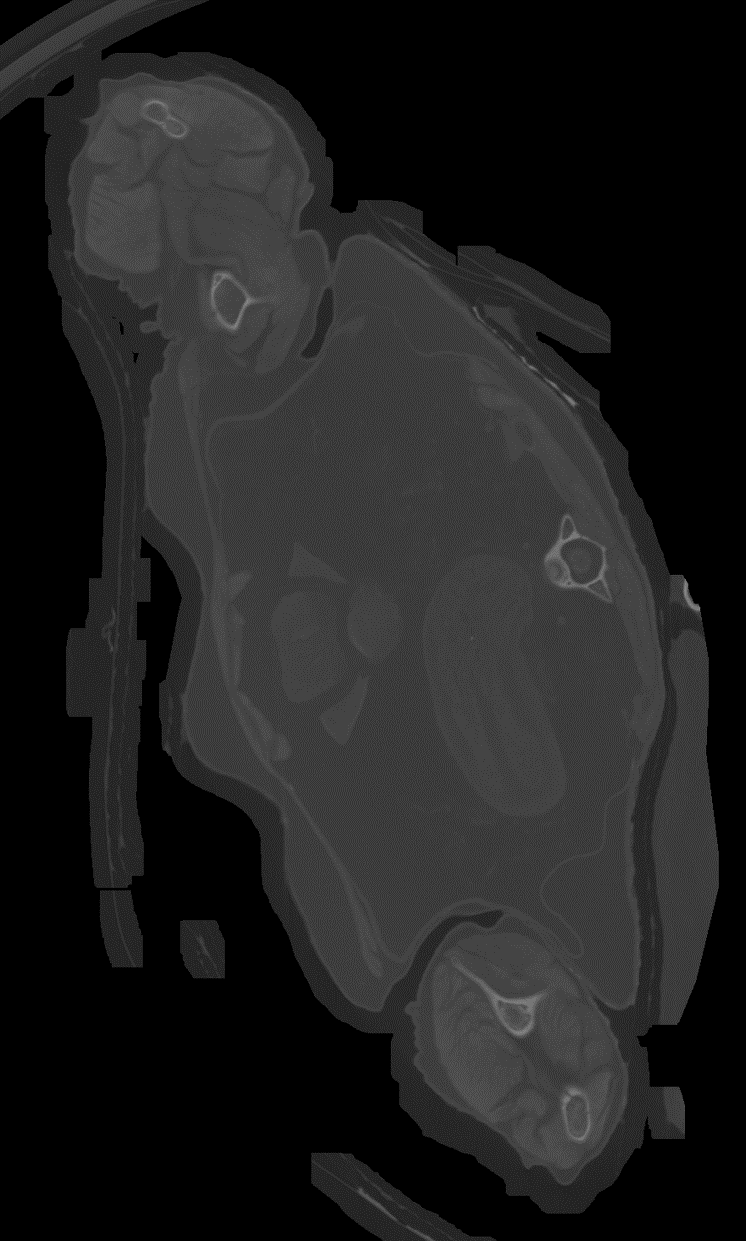

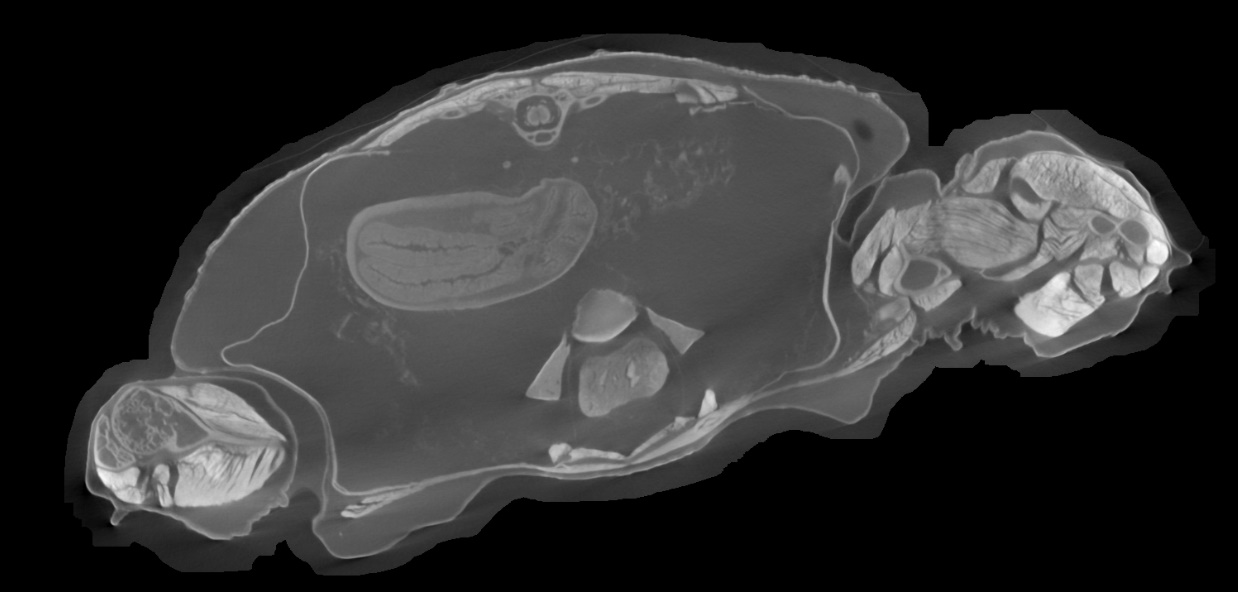


B)

A)

Figure S2 - Example of radiopacity before and after iodine staining. This specimen of *Ecnomiohyla miliaris* was stained and initially scanned four years prior (A) to the second scan (B).

Data normality

Table S1 – The data which do not have normally distributed residuals according to Shapiro-Wilk normality tests which are controlled for phylogeny (Revell, 2010). All data excluding muscle head counts have been log-transformed prior to testing. All remaining variables are normally distributed, including the total relative muscle mass for all hindlimb segments, the relative length of the dorsal pelvis crests, and the length of their associated pelvis muscle attachment sites.

|  | **W** | **p** |
| --- | --- | --- |
| **Muscle mass (relative to total segment mass)** | | |
| Longissimus dorsi | 0.833 | <0.001 |
| Coccygeoiliacus | 0.764 | <0.001 |
| Iliacus externus | 0.834 | <0.001 |
| Semimembranosus | 0.884 | 0.004 |
| Iliofibularis | 0.914 | 0.018 |
| Extensor brevis superhallucis | 0.913 | 0.018 |
| **Functional group (relative to total segment mass)** | | |
| Femur retraction | 0.907 | 0.012 |
| **Bone and muscle lengths (relative to snout-vent length)** | | |
| Urostyle | 0.895 | 0.006 |
| Coccygeoiliacus | 0.858 | <0.001 |
| Iliofibularis | 0.899 | 0.008 |
| Iliofemoralis | 0.892 | 0.005 |
| Adductor magnus (dorsal head) | 0.881 | 0.003 |
| **Muscle head count** | | |
| Pelvis | 0.773 | <0.001 |

Phylogenetic signal

The phylogenetic signal of each variable was extracted using the phylosig function in *phytools* (Revell, 2012). Then, fitContinuous was used to compare different models of evolution - a Brownian motion model, an Ornstein-Uhlenbeck model with a single optimum (i.e., identifies whether species are converging on one phenotype that is unrelated to phylogenetic history), the early-burst model (i.e., a model where the rate of evolution increases or decreases exponentially through time), and white noise (a non-phylogenetic model where there is no covariance structure among species).

Table S2 – The best to worst evolutionary models for all size-corrected variables with evidence of a phylogenetic signal (lambda) – Brownian motion (BM), white noise (WN), early burst (EB), lambda (L) and Ornstein-Uhlenbeck with a single optimum (OU). The *p-*value refers to how confident we can be that the phylogenetic signal is significantly different from zero - significant phylogenetic signals are highlighted in bold. This table includes the log-likelihood, AIC corrected for small sample size (AICc), and the AIC weight for the best model. Any data not included in this table had a phylogenetic signal of zero, with a *p-*value of one.

|  | **Data** | **Lambda** | ***p*-value** | **Best to worst model** | **Best model logLikelihood** | **Best model AICc** | **Best model AICc weight** |
| --- | --- | --- | --- | --- | --- | --- | --- |
| **Muscle head count** | Pelvis | 1 | 0.316 | BM, WN, EB, L/OU | -11.33 | 26.67 | 0.361 |
|  | Thigh | 0.716 | 0.178 | BM, WN, L, OU, EB | -51.84 | 107.67 | 0.367 |
| **Functional group mass** | Femur protraction and adduction | 0.906 | 0.187 | BM, WN, L, EB, OU | 9.05 | -14.1 | 0.393 |
|  | Femur retraction and adduction | 0.446 | 0.345 | WN, BM, OU, L, EB | 4.19 | -4.38 | 0.274 |
|  | Femur protraction and abduction | 1 | 0.079 | BM, EB, OU, L, WN | -1.025 | 6.05 | 0.428 |
|  | Knee flexion | 0.761 | 0.193 | BM, WN, L, OU, EB | -20.39 | 44.79 | 0.378 |
|  | Knee extension | 1 | 0.234 | BM, WN, L/OU/EB | 2.144 | -0.288 | 0.385 |
| **Pelvis muscle mass** | Coccygeosacralis | **1** | **0.043** | BM, EB, L/OU, WN | -45.34 | 94.67 | 0.445 |
| **Thigh muscle mass** | Iliacus internus | 1 | 0.079 | BM, EB, L/OU, WN | -1.025 | 6.05 | 0.428 |
|  | Tensor fascia latae | 0.766 | 0.12 | BM, OU, L, EB, WN | -12.02 | 28.04 | 0.386 |
|  | Cruralis | 0.928 | 1 | WN, BM, OU, L, EB | -3.48 | 10.95 | 0.343 |
|  | Gluteus magnus | 0.290 | 0.67 | WN, L, OU, BM, EB | -2.57 | 9.14 | 0.44 |
|  | Gracilis major + gracilis minor | 0.47 | 0.282 | BM, WN, OU, L, EB | 3.04 | -2.09 | 0.276 |
|  | Semitendinosus | 0.761 | 0.193 | BM, WN, L, OU, EB | -20.39 | 44.79 | 0.378 |
| **Shank muscle mass** | Extensor cruris brevis | 0.649 | 0.49 | BM, WN, L, OU, EB | -22.75 | 49.51 | 0.32 |
| **Tarsal muscle mass** | Intertarsalis | 0.578 | 0.702 | WN, BM, OU, L, EB | -13.29 | 30.59 | 0.315 |
|  | Adductor brevis dorsalis V | **1** | **0.003** | EB, BM, L/OU, WN | -5.02 | 16.03 | 0.524 |
| **Bone length** | Calcaneus  Ilium | 0.266  0.999 | 0.688  0.091 | WN, BM, OU, L, EB  BM, EB, L/OU, WN | 50.47  56.99 | -96.93  -109.98 | 0.393  0.423 |
| **Crest length** | Ilium  Urostyle | 0.999  0.999 | **0.013**  **0.009** | BM, EB, OU, L, WN  BM, EB, OU, L, WN | -2.64  3.62 | 9.727  -2.79 | 0.463  0.423 |
| **Pelvis muscle length** | Longissimus dorsi  Coccygeosacralis  Coccygeoiliacus  Iliacus externus | 0.601  0.999  0.453  0.999 | 0.351  0.094  0.406  0.066 | BM, WN, L, OU, EB  BM, EB, L/OU, WN  WN, BM, L, OU, EB  BM, EB, OU, L, WN | 38.60  37.89  36.80  48.25 | -73.19  -71.79  -69.60  -92.49 | 0.31  0.424  0.31  0.341 |
| **Thigh muscle length** | Adductor magnus (dorsal head)  Semitendinosus (dorsal head) | 0.754  0.498 | 0.428  0.387 | BM, WN, L, OU, EB  WN, BM, L, OU, EB | 33.61  32.64 | -63.23  -61.29 | 0.338  0.316 |
| **Tarsal muscle length** | Adductor brevis dorsalis V | 0.17 | 0.765 | WN, OU, L, BM, EB | 62.78 | -121.56 | 0.464 |

**Relative muscle lengths**

Once corrected for body size, muscle length was compared to the associated pelvis and hindlimb bone length to determine whether bone length is a suitable proxy for estimating muscle size, which would have important implications for studies which infer behaviour from fossils (Table S3; Figures S3 to S6).

**Figure S3** - The length of longissimus (LD), coccygeosacralis (CS), coccygeoiliacus (CI) and iliacus externus (IE) relative to snout-vent length (SVL) across locomotor modes. The pyriformis is excluded since it originates at the posterior tip of the urostyle and inserts onto the thigh. The error bars represent standard deviation.

**Table S3** – The significant differences between locomotor modes in the length of each bone and muscle relative to snout-vent length according to a phylogenetic ANOVA. Any variables and locomotor pairing not listed did not show significant differences, e.g., pelvis muscles. The first locomotor mode described in each pairing has the longer bone/muscle even after the *p-*value has been adjusted for multiple testing using a Bonferroni correction. The full name of each muscle is provided in Figures 1 and 2 and Table 1 of the main text.

| **Variable** | **Sum of Squares** | **Mean Square** | **F-value** | ***p-*value** | **Pairs** |
| --- | --- | --- | --- | --- | --- |
| **Relative bone length** | | | | | |
| Femur | 0.048 | 0.012 | 8.289 | 0.001 | AJ vs BWH  AQ vs BWH  TJ vs BWH |
| Tibiofibula | 0.074 | 0.018 | 7.472 | 0.002 | All LMs vs BWH |
| Calcaneum | 0.034 | 0.008 | 7.831 | 0.001 | AJ vs BWH  TJ vs BWH  WH vs BWH |
| **Relative thigh muscle length** | | | | | |
| CR | 0.037 | 0.009 | 5.789 | 0.002 | AJ vs BWH  AQ vs BWH  TJ vs BWH |
| SM | 0.033 | 0.008 | 6.242 | 0.002 | AJ vs BWH  AQ vs BWH  TJ vs BWH |
| QF | 0.017 | 0.004 | 3.033 | 0.023 | WH vs AQ |
| SA | 0.047 | 0.012 | 5.075 | 0.002 | AQ vs BWH  TJ vs BWH |
| AMv | 0.051 | 0.013 | 9.818 | 0.001 | AQ vs BWH  AJ vs WH  AQ vs BWH  TJ vs BWH |
| GRM | 0.033 | 0.008 | 5.996 | 0.001 | None |
| AL | 0.15 | 0.037 | 2.967 | 0.031 | AQ vs WH |
| **Relative shank muscle length** | | | | | |
| PL | 0.075 | 0.019 | 10.522 | 0.001 | AJ vs BWH  AQ vs BWH  TJ vs BWH |
| TiP | 0.073 | 0.018 | 5.743 | 0.002 | AQ vs BWH  AJ vs WH |
| PER | 0.087 | 0.022 | 10.153 | 0.001 | AJ vs BWH  AQ vs BWH  TJ vs BWH |
| ECB | 0.033 | 0.008 | 5.312 | 0.002 | AJ vs BWH  AQ vs BWH |
| TiAB | 0.03 | 0.007 | 4.513 | 0.004 | AJ vs BWH  AQ vs BWH |
| TiAL | 0.066 | 0.016 | 7.226 | 0.001 | AJ vs BWH  TJ vs BWH |
| **Relative proximal foot muscle length** | | | | | |
| PP | 0.031 | 0.008 | 12.029 | 0.001 | AJ vs AQ  AJ vs BWH  AJ vs TJ  TJ vs BWH  WH vs BWH |
| TaP | 0.023 | 0.006 | 5.397 | 0.004 | AJ vs BWH |
| TaA | 0.012 | 0.003 | 3.641 | 0.013 | AJ vs BWH  TJ vs BWH |
| EDCL | 0.038 | 0.01 | 3.275 | 0.014 | AJ vs BWH  AQ vs BWH  WH vs BWH |
| FDBS | 0.024 | 0.006 | 8.096 | 0.001 | AJ vs BWH  WH vs BWH |
| INT | 0.019 | 0.005 | 5.377 | 0.001 | AJ vs BWH  TJ vs BWH  WH vs BWH |
| AbdV | 0.012 | 0.003 | 5.449 | 0.002 | AJ vs BWH  AJ vs WH  TJ vs BWH |

Figure S4 - The length of each muscle in the thigh relative to the length of the femur, colour coded by locomotor mode. The full names of each muscle can be found in Table 1 of the main text. Error bars represent standard deviation.

Figure S5 - The length of each muscle in the shank relative to the length of the tibiofibula, colour coded by locomotor mode. The full names of each muscle can be found in Table 1 of the main text. Error bars represent standard deviation.

**Figure S6** - The length of each muscle in the proximal foot relative to the length of the calcaneus, colour coded by locomotor mode. The full names of each muscle can be found in Figure 2 of the main text. Error bars represent standard deviation.

##### pPCA loadings

Table S4 - pPCA loadings from the first four axes for the size-corrected pelvis, thigh, shank, and tarsal muscle masses. For each PC axis, light and dark boxes highlight the largest positive and negative loadings respectively. Abbreviations can be found in Table 1, Figure 1, and Figure 2 of the main text.

| **Axis** | **PC1** | **PC2** | **PC3** | **PC4** |
| --- | --- | --- | --- | --- |
| Pelvis | | | | |
| Total variance explained (%) | 50.04 | 24.73 | 11.34 | 9.79 |
| LD | 0.591 | 0.687 | -0.406 | -0.112 |
| CGS | -0.933 | 0.352 | 0.019 | -0.059 |
| CGI | -0.186 | -0.717 | -0.379 | -0.536 |
| PY | -0.389 | -0.292 | -0.63 | 0.603 |
| IE | 0.552 | 0.153 | 0.669 | 0.338 |
| Thigh | | | | |
| Total variance explained (%) | 30.38 | 17.18 | 12.05 | 11.32 |
| II | 0.563 | 0.003 | 0.277 | -0.18 |
| TFL | -0.225 | 0.481 | 0.139 | -0.628 |
| CR | -0.564 | -0.534 | -0.401 | 0.094 |
| GM | -0.628 | 0.236 | 0.055 | -0.219 |
| SM | 0.12 | 0.408 | 0.63 | 0.208 |
| Ifib | 0.431 | 0.684 | -0.305 | -0.251 |
| Ifem | 0.374 | 0.402 | 0.642 | -0.369 |
| hip_muscles | 0.549 | -0.336 | 0.211 | 0.457 |
| SA | -0.315 | 0.727 | -0.251 | 0.086 |
| AM | -0.393 | 0.273 | 0.581 | -0.042 |
| GRM + GRm | -0.07 | -0.009 | -0.019 | 0.391 |
| PT + AL | 0.818 | -0.167 | -0.352 | -0.178 |
| ST | 0.725 | 0.245 | 0.149 | 0.425 |
| Shank | | | | |
| Total variance explained (%) | 49.3 | 22.88 | 16.6 | 7.03 |
| PL | -0.381 | -0.521 | 0.678 | -0.072 |
| TiP | 0.644 | 0.211 | -0.034 | -0.439 |
| PER | -0.701 | 0.283 | -0.58 | 0.278 |
| ECB | 0.92 | -0.012 | 0.128 | 0.366 |
| TiAB | -0.027 | 0.948 | 0.312 | -0.013 |
| TiAL | 0.81 | 0.148 | -0.51 | -0.177 |
| Tarsals | | | | |
| Total variance explained (%) | 37.01 | 29.28 | 10.49 | 9.4 |
| PP | -0.413 | 0.029 | 0.854 | 0.058 |
| TaP | -0.339 | 0.25 | 0.186 | 0.495 |
| TaA | 0.461 | 0.036 | -0.148 | -0.204 |
| EBS | -0.449 | -0.889 | -0.075 | -0.047 |
| EDCL | -0.883 | 0.42 | -0.146 | -0.146 |
| FDBS | 0.271 | -0.009 | -0.367 | 0.487 |
| INT | 0.435 | -0.021 | 0.216 | -0.819 |
| AbdV | 0.482 | 0.004 | -0.491 | -0.189 |

**Dorsal crests on pelvis bones**

**Table S5 -** Phylogenetic least squares (PGLS) models examining whether there are correlations between the mass of the pelvis muscles and the length of their attachment site on their associated bones (excluding the crest, if present), and their dorsal crests. Significant values have been highlighted in bold.

| **Muscle mass** | **Attachment site** | **t-value** | ***p*-value** |
| --- | --- | --- | --- |
| Longissimus dorsi | Urostylic crest | 1.556 | 0.131 |
| Coccygeosacralis | Urostylic crest | 6.68 | **<0.001** |
| Coccygeoiliacus | Urostylic crest | 1.522 | 0.139 |
| Coccygeoiliacus | Iliac crest | 1.899 | 0.068 |
| Iliacus externus | Iliac crest | -1.011 | 0.321 |
| Longissimus dorsi | Urostyle | 2.13 | **0.042** |
| Coccygeosacralis | Urostyle | 4.237 | **<0.001** |
| Coccygeoiliacus | Urostyle | 4.277 | **<0.001** |
| Coccygeoiliacus | Ilium | 3.583 | **0.001** |
| Iliacus externus | Ilium | 1.371 | 0.181 |

**Testing if scan resolution is a confounding variable**

Previous studies have suggested that scan resolution may be a confounding variable that could impact the data obtained from CT scans (Broeckhoven & Plessis, 2018). Poor scan resolution can make it difficult to visualise muscle boundaries and can cause overestimations in object size, especially when examining small specimens. Therefore, voxel size, the pixel dimensions in each plan of view, were included as an explanatory factor in all the ANOVA and least-squares models to see if low scan resolution might be correlated with muscle volumes (Table S6) or muscle head count (Table S7). The best model is determined by the lowest Akaike Information Criterion value, which estimates how well models fit the data they were generated from.

Table S6 - Table of Akaike Information Criterion (AIC) for each analysis of variance (ANOVA). Rows highlighted in bold show that that adding voxel size as an explanatory variable for muscle composition across all hindlimb segments did not result in a better fit of the data.

| **ANOVA model** | **df** | **AIC** |
| --- | --- | --- |
| Total thigh mass ~ LM + voxel size | 10 | -68.56 |
| **Total thigh mass ~ LM** | **9** | **-70.48** |
| Total shank mass ~ LM + voxel size | 10 | -15.46 |
| **Total shank mass ~ LM** | **9** | **-17.43** |
| Total tarsal mass ~ LM + voxel size | 10 | 9.64 |
| **Total tarsal mass ~ LM** | **9** | **7.77** |

Table S7 - Table of Akaike Information Criterion (AIC) for each model of muscle head count across the pelvis, thigh, and shank. All phylogenetic least squares (PGLS) models show a better fit than ordinary least squares (OLS) models, meaning that phylogenetic history is an important explanatory variable. Rows highlighted in bold show that the model fits the data best when locomotor mode was also not incorporated into the model.

|  | **Model** | **df** | **AIC** |
| --- | --- | --- | --- |
| PGLS | Pelvis count ~ LM + voxel size | 6 | 33.17 |
|  | Pelvis count ~ LM | 5 | 31.38 |
|  | Pelvis count ~ voxel size | 2 | 26.6 |
|  | **Pelvis count ~ 1** | **1** | **24.65** |
|  | Thigh count ~ LM + voxel size | 6 | 107.32 |
|  | Thigh count ~ LM | 5 | 106.03 |
|  | Thigh count ~ voxel size | 2 | 104.44 |
|  | **Thigh count ~ 1** | **1** | **104.28** |
|  | Shank count ~ LM + voxel size | 6 | 35.26 |
|  | **Shank count ~ LM** | **5** | **33.41** |
|  | Shank count ~ voxel size | 2 | 34.74 |
|  | **Shank count ~ 1** | **1** | **33.18** |
| OLS | Pelvis count ~ LM + voxel size | 7 | 35.82 |
|  | Pelvis count ~ LM | 6 | 34.08 |
|  | Thigh count ~ LM + voxel size | 7 | 113.02 |
|  | Thigh count ~ LM | 6 | 111.75 |
|  | Shank count ~ LM + voxel size | 7 | 37.26 |
|  | Shank count ~ LM | 6 | 35.41 |

**Supplementary Dataset**

#### Available to download from DOI:10.6084/m9.figshare.26357395.

#### ‘Study taxa’

This dataset contains all the information for each specimen used, including locomotor mode, habitat type, scanning parameters, staining protocols, and sources.

#### ‘Skeletal data’

Raw length measurements for each of the post-vertebral bones.

**‘Pelvic crest data’**

#### Raw length measurements for the dorsal crests on the ilia and urostyle, as well as the length of the length of attachment sites for their associated muscles.

#### ‘Muscle data’

Raw length and volume measurements for each of the post-vertebral muscles, excluding the distal foot.

#### ‘Muscle head counts’

The number of separate muscle heads observed in the pelvis, thigh and shank for the specimens used. Muscle abbreviations can be found in Table 1, Figure 1, and Figure 2 of the main text. Quotes containing extra information and colouring are based on Přikryl *et al.* (2009). Green indicates that our findings match, red indicates that they don't. Orange means there is nothing to say this is not true, or there is suggestive wording in Přikryl, e.g., 'may be absent/separate heads in some individuals/species of X.' Colouring is mainly used for when the number of muscle heads are different from the usual.
